# Quantification of [^11^C]CURB PET using an irreversible reference tissue model with a cluster-derived pseudo-reference region

**DOI:** 10.3389/fnins.2026.1882674

**Published:** 2026-07-20

**Authors:** Lucas Narciso, Shahtaj S. Dheda, Raesham Mahmood, Rachel F. Tyndale, Tina McCluskey, Jerry Warsh, Bernard Le Foll, Kimberly L. Desmond, Stefan Kloiber, Isabelle Boileau

**Affiliations:** 1 Brain Health Imaging Centre, Centre for Addiction and Mental Health, Toronto, ON, Canada; 2 Department of Psychiatry, University of Toronto, Toronto, ON, Canada; 3 Institute of Medical Science, Temerty Faculty of Medicine, University of Toronto, Toronto, ON, Canada; 4 Department of Pharmacology and Toxicology, University of Toronto, Toronto, ON, Canada; 5 Campbell Family Mental Health Research Institute, Centre for Addiction and Mental Health, Toronto, ON, Canada; 6 Translational Addiction Research Laboratory, Centre for Addiction and Mental Health, Toronto, ON, Canada; 7 Department of Family and Community Medicine, University of Toronto, Toronto, ON, Canada; 8 Waypoint Centre for Mental Health Care, Waypoint Research Institute, Penetanguishene, ON, Canada

**Keywords:** [^11^C]CURB, fatty acid amide hydrolase (FAAH), irreversible reference tissue model (IRTM), non-invasive quantification, positron emission tomography (PET)

## Abstract

**Introduction:**

Quantification of [^11^C]CURB, an irreversible positron emission tomography (PET) radiopharmaceutical used to image fatty acid amide hydrolase (FAAH), requires invasive arterial sampling. Developing a non-invasive approach for [^11^C]CURB is challenging, since traditional reference region models are not directly applicable and the ubiquitous brain expression of FAAH complicates the identification of a ligand-free reference region. This study aimed to introduce and validate the irreversible reference tissue model (IRTM) used in conjunction with a data-driven, clustering-based white matter (WM) pseudo-reference region.

**Methods:**

IRTM was implemented using a coupled-fit approach to estimate a common $k_{2}^{{}^{\prime }}$, resulting in two independent parameters per volume-of-interest: *R_1_* and *k_f_*. Primary quantification was performed using the macroparameters $R_{i}=K_{i}/K_{1}^{\prime }=R_{1}\,k_{3}/k_{f}$ (relative net influx) and $R\,k_{3}=\lambda \,k_{3}/K_{1}^{\prime }=R_{1}\,k_{3}/k_{2}$ (relative trapping index). Simulations characterized the sensitivity of IRTM to differences in cerebral blood volume between target and reference tissues. The model was validated against the gold standard arterial input function (AIF)-based quantification using retrospective [^11^C]CURB PET data from 10 healthy participants. A partition-based clustering algorithm was used to extract the centermost WM time-activity curve as a pseudo-reference region.

**Results:**

In the IRTM validation study with human data, *R_i_* emerged as a more robust metric than *Rk*_3_, with the latter exhibiting high between-subject variability. Although simulations identified systematic biases in IRTM estimates driven by blood volume asymmetries, IRTM-derived *R_i_* estimates showed strong concordance with AIF-based *R_i_* (*R*^2^ = 0.96), λ*k*_3_ (*R*^2^ = 0.90), and *K_i_* (*R*^2^ = 0.96). Mean errors in *R_i_* ranged from −11.2 ± 9.9% in the frontal lobe to −3.1 ± 10.4% in the amygdala. Voxel-wise parametric maps demonstrated high image quality and signal-to-noise ratio at the individual subject level.

**Conclusion:**

This study provides a validated, fully non-invasive framework for [^11^C]CURB PET quantification. Combined with a partition-based clustering approach to select a data-driven WM pseudo-reference region, IRTM achieved excellent concordance with AIF-based measurements, supporting the feasibility of IRTM for non-invasive FAAH imaging.

## Introduction

The endocannabinoid system (ECS) plays a critical role in modulating neurotransmitter release and is involved in a wide range of physiological and cognitive processes, including the modulation of pain, synaptic plasticity, inflammation, and emotional and behavioral regulation (Katona and Freund, 2012; Mechoulam and Parker, 2013). Dysregulation of the ECS has been implicated in several neuropsychiatric and neurological diseases, including post-traumatic stress disorder (Mayo et al., 2022), schizophrenia (Lu and Mackie, 2021), eating disorders (Bourdy and Befort, 2023), addiction (Sloan et al., 2017; Poluga et al., 2026), and neurodegenerative diseases such as Alzheimer’s disease and Parkinson’s disease (Basavarajappa et al., 2017). While alterations in the ECS can manifest across its complex network of cannabinoid receptors, endogenous ligands, and metabolic pathways, the fatty acid amide hydrolase (FAAH) is a key target of interest. FAAH is localized predominantly at the postsynaptic neurons, where it is associated with intracellular membranes in somatodendritic compartments. There it hydrolyzes the endocannabinoid anandamide (N-arachidonoylethanolamide) and related fatty acid amides, thereby terminating cannabinoid signaling (Cravatt et al., 1996; Piomelli, 2003).

Positron emission tomography (PET) imaging with [^11^C]CURB offers *in vivo* quantification of FAAH density (Boileau et al., 2015a; Varlow et al., 2020). To this date, quantification of [^11^C]CURB PET has required measuring the arterial input function (AIF) (Rusjan et al., 2013; De Pol et al., 2025; Rafiei et al., 2025). However, challenges associated with arterial sampling continue to limit the clinical translation of quantitative PET imaging, motivating the development of non-invasive alternatives (van der Weijden et al., 2023). Removing the requirement for arterial sampling simplifies experimental procedures, reduces risk and participant burden, and improves recruitment and retention, particularly in vulnerable populations. Non-invasive quantification also increases the scalability and feasibility of PET imaging in larger cohorts and multi-center studies. Furthermore, recent advances in PET instrumentation (Lecomte et al., 2023; Li et al., 2024) provide incentives to revisit and refine reference tissue-based modeling approaches. The only strategy previously proposed for the non-invasive quantification of [^11^C]CURB was the use of a population-based input function (Narciso et al., 2024a) to replace individual arterial sampling with a scaled average AIF. While this strategy substantially reduces invasiveness and has demonstrated good performance at the group level, its accuracy depends on the validity of population-level assumptions regarding tracer delivery and metabolism.

The Gjedde-Patlak graphical analysis with reference tissue provides an alternative non-invasive framework for irreversible radiopharmaceuticals (Patlak and Blasberg, 1985). By assuming a uniform non-displaceable volume of distribution across brain regions, the resulting slope ($K_{i}^{r\,e\,f}$) represents the net clearance-normalized influx rate ($K_{i}^{r\,e\,f}=k_{2}\,k_{3}/\left(k_{2}+k_{3}\right)$, where *k_2_* and *k_3_* are the efflux and irreversible trapping rate constants, respectively). However, based on microparameters previously reported (Rusjan et al., 2013), [^11^C]CURB $K_{i}^{r\,e\,f}$ is estimated to be notably low (approx. 0.04 min^–1^). This proximity to zero introduces statistical bias and uncertainty because the error distribution becomes non-Gaussian as estimates approach the zero-boundary. Consequently, this behavior results in numerical instability and reduces sensitivity, limiting the practical utility of reference Gjedde-Patlak for FAAH quantification with [^11^C]CURB PET. The transport-limited reference tissue model (Herholz et al., 2001; Nagatsuka et al., 2001) was another approach developed for radiotracers without a traditional reference region, but with a “positive” reference region of very rapid irreversible binding or metabolism (i.e., $k_{3}^{\prime }\gg k_{2}^{\prime }$; where $k_{2}^{\prime }$ and $k_{3}^{\prime }$ are the reference *k_2_* and *k_3_* rate constants, respectively); however, this assumption does not hold for [^11^C]CURB. While the aforementioned models address irreversible kinetics, the most widely used non-invasive approach is the simplified reference tissue model (SRTM) (Lammertsma and Hume, 1996) that relies on the availability of a reference region with negligible specific binding (i.e., its kinetics reflects non-displaceable uptake).

FAAH biodistribution has been extensively characterized in both rodent and human brains using a combination of immunocytochemistry with affinity-purified antibodies, enzymatic activity assays, and *in situ* hybridization (Thomas et al., 1997; Egertová et al., 1998; Tsou et al., 1998; Romero et al., 2002). In both species, the highest FAAH expression occurs in the neocortex, hippocampus, amygdala, cerebellum, and selected subcortical nuclei (Thomas et al., 1997; Tsou et al., 1998; Romero et al., 2002). While predominantly expressed in neuronal somata and dendrites, FAAH is also localized in white matter (WM) macroglia (primarily astrocytes and mature oligodendrocytes), albeit at substantially lower levels (Romero et al., 2002). Thus, WM represents a candidate reference region for [^11^C]CURB; however, disease-related changes in FAAH expression could violate modeling assumptions and introduce quantification bias, requiring adequate validation for the specific study population (Núñez et al., 2008).

In recent years, WM has been proposed as a reference region for various radiotracers with a prevalence of cortical or sub-cortical gray matter (GM) binding (Wong et al., 2010; Brendel et al., 2015; Lowe et al., 2018; Uribe et al., 2026). However, careful selection of the WM volume-of-interest (VOI) is required to minimize GM spillover. As a result, atlas-based WM VOI definition can introduce quantification biases (Rossano et al., 2020). Data-driven approaches, such as partition-based clustering, offer a powerful alternative by identifying WM regions based on kinetic similarity rather than anatomy (Ashburner et al., 1996). Beyond VOI definition, the assumptions regarding cerebral blood volume (*V_b_*) present a further challenge. A core premise of SRTM is that intravascular signal contribution to both target and reference regions are negligible; however, Salinas et al. demonstrated that a *V_b_* of 0.05 mL/cm^3^ (representative of GM) can introduce a ∼5% bias in SRTM-derived non-displaceable binding potential (*BP*_*ND*_). Given that blood-borne signal is lower in WM than GM (Leenders et al., 1990), using a WM reference region introduces a *V_b_* asymmetry that may further violate assumptions and compromise quantification accuracy.

By design, SRTM is not directly applicable to irreversible radiotracers as it relies on *BP*_*ND*_ (*k*_3_/*k*_4_; where *k_4_* is the dissociation rate constant) as a core fitting parameter. Because *k_4_* = 0 for irreversible radiotracer, *BP*_*ND*_ becomes mathematically undefined, motivating the development of a model tailored to irreversible target kinetics that utilizes a reference region that follows the one-tissue compartmental model (1TCM). This led to the formulation of the irreversible reference tissue model (IRTM) described in this work. In the context of [^11^C]CURB, although no true reference region devoid of FAAH exists in the brain, the lower concentration of FAAH expression in WM (Benito et al., 2007) supports its use as a pseudo-reference region for non-invasive quantification.

This study was aimed to establish a robust, non-invasive framework for the quantification of FAAH density using [^11^C]CURB PET. Our objectives were fourfold. First, to derive the theoretical framework for IRTM, a reference tissue model specifically adapted for irreversible radiotracers. Second, to implement a data-driven, partition-based clustering algorithm to objectively identify a WM pseudo-reference region. Third, to perform simulations to characterize the impact of blood volume effects on IRTM parameter estimation. Lastly, to validate IRTM against AIF-based quantification using retrospective [^11^C]CURB PET data acquired from healthy participants. Collectively, these analyses aim to delineate the conditions under which IRTM provides reliable quantitative parameter estimates for [^11^C]CURB PET and to establish best practices for reference region selection in the absence of arterial sampling.

## Materials and methods

### Theory

**FIGURE 1:**
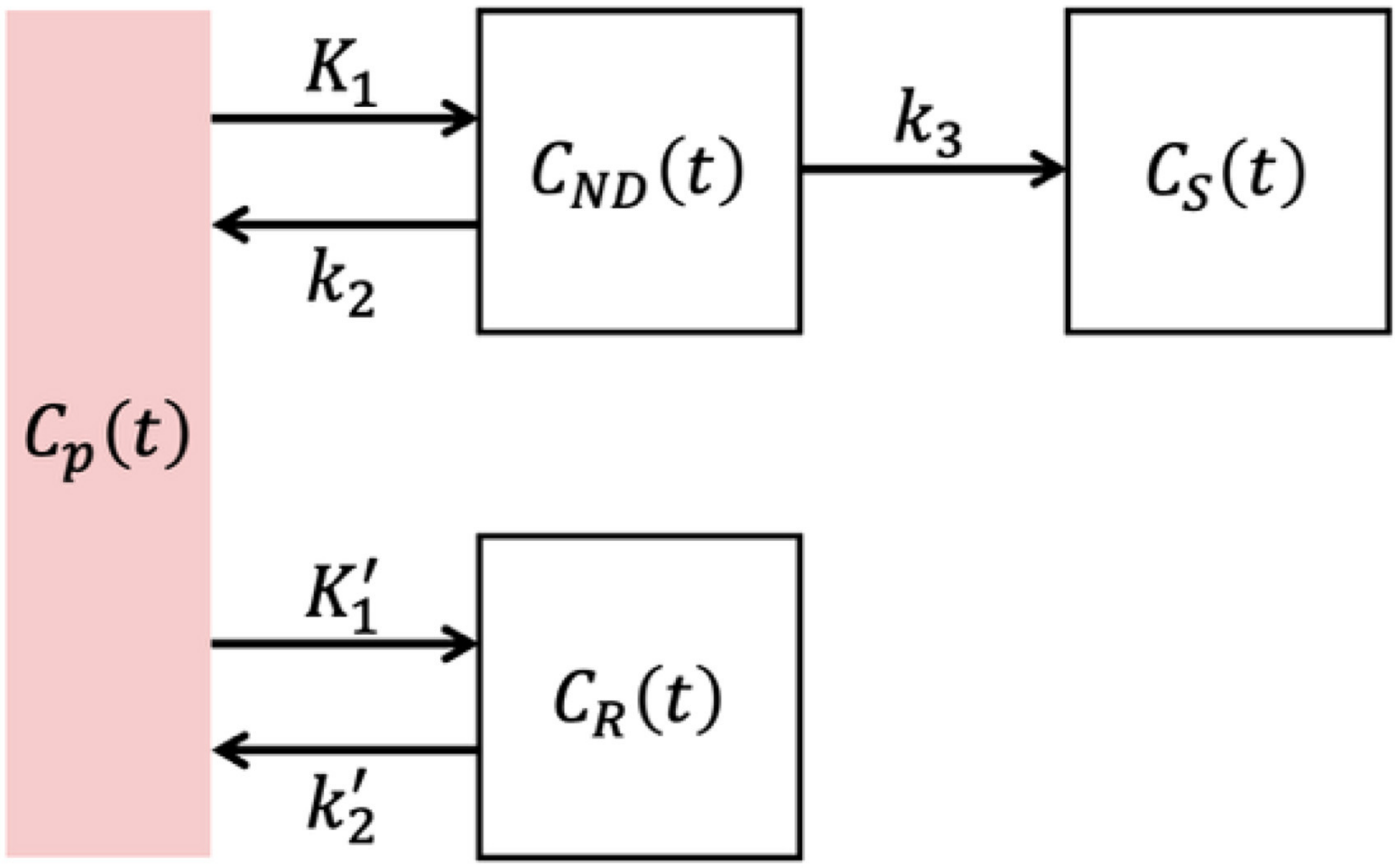
Compartmental model and derivation of IRTM. [^11^C]CURB follows irreversible kinetics and is described by the i2TCM, in which the time-varying activity concentrations in the non-displaceable [*C*_*ND*_(*t*)] and specific [*C*_*S*_(*t*)] compartments are governed by the rate constants *K*_1_ (i.e., the influx rate constant; mL/cm^3^/min), *k*_2_ (the efflux rate constant; min^–1^), and *k*_3_ (the irreversible trapping rate constant, related to FAAH enzymatic activity; min^–1^). *C*_*p*_(t) denotes the activity concentration of [^11^C]CURB in plasma. Assuming the existence of a reference region of activity concentration *C*_*R*_(t), described by the 1TCM with rate constants *K*1′ and *k*2′. Solving the model equations yields the analytical expression of the IRTM (Equation 10).


[^11^C]CURB is a radiotracer best described by irreversible kinetics and is well characterized by the i2TCM (Figure 1), governed by Equations (1) and (2).

$$\frac{d\,C_{N\,D}\,\left(t\right)}{d\,t}=K_{1}\,C_{p}\,\left(t\right)-\left(k_{2}+k_{3}\right)\,C_{N\,D}\,\left(t\right)$$ (1)

$$\frac{d\,C_{S}\,\left(t\right)}{d\,t}=k_{3}\,C_{N\,D}\,\left(t\right)$$ (2)

where *K_1_* (mL/cm^3^/min) is the plasma-to-tissue influx rate constant into the non-displaceable (ND) compartment, *k_2_* (min^–1^) is the efflux rate constant from the ND compartment back to plasma, and *k_3_* (min^–1^) is the irreversible trapping rate constant associated with FAAH enzymatic activity. *C*_*p*_(*t*), *C*_*ND*_(*t*), and *C*_*S*_(*t*) denote the activity concentration of [^11^C]CURB in plasma, in the ND compartment, and in the irreversibly bound specific (S) compartment, respectively.

Applying the Laplace transform to Equations (1) and (2), and defining the total activity concentration as *C*_*T*_(*t*) = *C*_*ND*_(*t*) + *C*_*S*_(*t*) yields the following expressions in the Laplace domain:


$$\bar{C_{N\,D}}\,\left(s\right)=\frac{K_{1}}{s+k_{f}}\,\bar{C_{p}}\,\left(s\right)$$ (3)

$$\bar{C_{S}}\,\left(s\right)=\frac{k_{3}}{s}\,\bar{C_{N\,D}}\,\left(s\right)=\left(\frac{k_{3}}{s}\right)\,\left(\frac{K_{1}}{s+k_{f}}\right)\,\bar{C_{p}}\,\left(s\right)$$ (4)

$$\bar{C_{T}}\,\left(s\right)=\left(\frac{s+k_{3}}{s}\right)\,\left(\frac{K_{1}}{s+k_{f}}\right)\,\bar{C_{p}}\,\left(s\right)$$ (5)

where *k*_*f*_ = *k*_2_ + *k*_3_ and *s* is the Laplace variable.

Next, assume the existence of a reference region with activity concentration *C*_*R*_(*t*) that follows the 1TCM (Equation 6), with $K_{1}^{\prime }$ and $k_{2}^{\prime }$ denoting the influx and efflux rate constants of the reference region, respectively. Similarly, Equation (6) can be rewritten as Equation (7) using the Laplace transform. Substituting Equation (7) into Equation (5) allows the total tissue activity concentration to be expressed in terms of *C*_*R*_(*t*)Equation (8).


$$\frac{d\,C_{R}\,\left(t\right)}{d\,t}=K_{1}^{\prime }\,C_{p}\,\left(t\right)-k_{2}^{\prime }\,C_{R}\,\left(t\right)$$ (6)

$$\bar{C_{p}}\,\left(s\right)=\left(\frac{s+k_{2}^{\prime }}{K_{1}^{\prime }}\right)\,\bar{C_{R}}\,\left(s\right)$$ (7)

$$\bar{C_{T}}\,\left(s\right)=R_{1}\,\frac{\left(s+k_{2}^{\prime }\right)\,\left(s+k_{3}\right)}{s\,\left(s+k_{f}\right)}\,\bar{C_{R}}\,\left(s\right)$$ (8)

where *R_1_* is the relative delivery rate ($R_{1}=K_{1}/K_{1}^{\prime }$).

Using partial fraction decomposition (Equation 9) and applying the inverse Laplace transform yields the solution to IRTM (Equation 10).


$$\bar{C_{T}}\,\left(s\right)=R_{1}\,\left(1+\left(\frac{k_{2}^{\prime }\,k_{3}}{k_{f}}\right)\,\frac{1}{s}-k_{2}\,\left(\frac{k_{f}-k_{2}^{\prime }}{k_{f}}\right)\,\left(\frac{1}{s+k_{f}}\right)\right)\,\bar{C_{R}}\,\left(s\right)$$ (9)

$$C_{T}\,\left(t\right)=$$ (10)

$$R_{1}\,\left(C_{R}\,\left(t\right)+\frac{k_{2}^{\prime }\,k_{3}}{k_{f}}\,\int_{0}^{t}C_{R}\,\left(u\right)\,du-k_{2}\,\frac{k_{f}-k_{2}^{\prime }}{k_{f}}\,C_{R}\,\left(t\right)*e^{-k_{f}\,t}\right)$$

where * is the convolution operator.

Although the IRTM formulation contains three fitting parameters per VOI (*R_1_*, *k_2_* and *k_3_*), only two parameters are identifiable due to the intrinsic dependency between *k_2_* and *k_3_*. To resolve this identifiability issue, the microparameter *k_2_* was computed as $k_{2}=R_{1}\,k_{2}^{\prime }$ (i.e., $R_{1}=K_{1}/K_{1}^{\prime }=k_{2}/k_{2}^{\prime }$) based on the assumption the non-displaceable partition coefficient (*K*_1_/*k*_2_) is uniform across brain regions. Under this constraint, the model is parameterized by two independent parameters per VOI: *R_1_* and *k_f_*, with *k*_3_ = *k*_*f*_−*k*_2_. In addition, in the validation study, $k_{2}^{\prime }$ was treated as a common parameter across all VOIs (i.e., coupled fitting), resulting in a total of *2n+1* fitting parameters, where *n* denotes the number of VOI TACs inputted to the fitting algorithm.

PET quantification using IRTM was performed by means of relative net influx (*R_i_*; Equation 11) and relative trapping index (*Rk*_3_; Equation 12), defined to provide macroparameters that are conceptually analogous to *K_i_* (net influx rate constant) and λ*k*_3_ (trapping index; λ = *K*_1_/*k*_2_) used in arterial-based quantification. By normalizing to $K_{1}^{\prime }$, *R_i_* and *Rk*_3_ act as indices of FAAH density that are isolated from global fluctuations in cerebral blood flow, ensuring the observed differences in [^11^C]CURB binding reflect enzyme trapping rather than changes in perfusion.


$$R_{i}=\frac{K_{i}}{K_{1}^{\prime }}=\frac{R_{1}\,k_{3}}{k_{f}}$$ (11)

$$R\,k_{3}=\frac{\lambda \,k_{3}}{K_{1}^{\prime }}=\frac{R_{1}}{k_{2}}\,k_{3}$$ (12)

### Simulations

**FIGURE 2:**
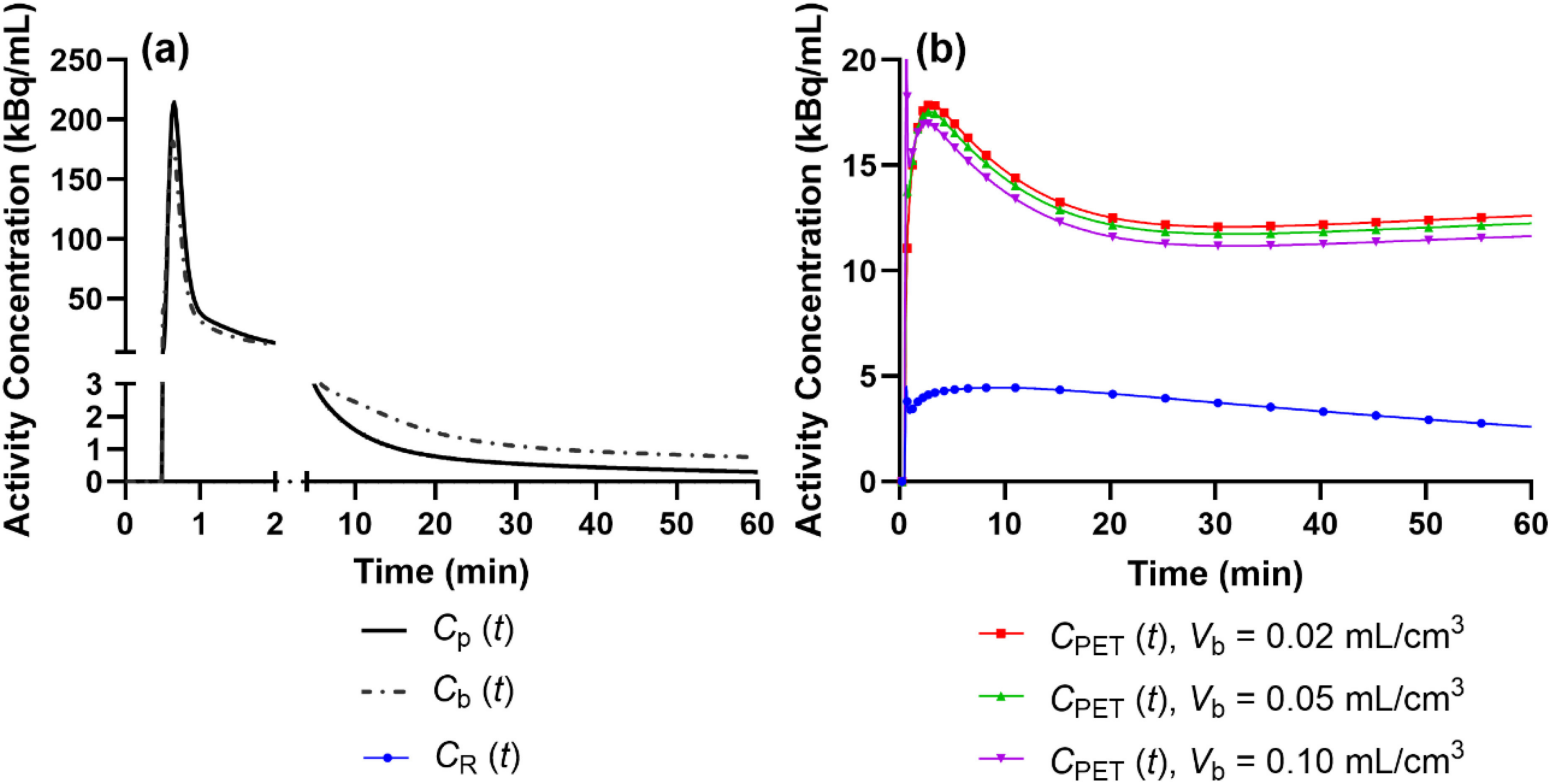
**(a)** Simulated plasma [*C*_*p*_(*t*); solid black line] and blood [*C*_*b*_(*t*); dot-dashed dark-gray line] AIFs, with simulation parameters estimated from observed mean [^11^C]CURB AIFs. The *x*-axis is split into 0–2 min and 4–60 min, and the *y*-axis is split into 0–3 kBq/mL and 5–250 kBq/mL, to better visualize the peaks and tails of the AIFs. **(b)** Examples of simulated PET TACs [*C*_*PET*_(*t*); *K*_1_ = 0.25 mL/cm^3^/min, *k*_2_ = 0.13 min^–1^, and *k*_3_ = 0.055 min^–1^] generated with *V*_*b*_ of 0.02 (red line, squares), 0.05 (green, triangles), and 0.10 mL/cm^3^ (purple, upside-down triangles). The reference TAC [*C*_*R*_(*t*); blue, circles] includes a blood volume fraction of 0.02 mL/cm^3^. Symbols correspond to PET reconstruction schedule.


A theoretical plasma AIF [*C*_*p*_(*t*); Figure 2a] was generated by combining six gamma-distribution functions according to Equation (13), with orders *k* = 7, 3, 3, 1, 0, and 0. The corresponding amplitude parameters *a_1_* to *a_6_* (2.1 × 10^1^ kBq/mL/min^7^, 6.5 × 10^5^ kBq/mL/min^3^, 6.5 × 10^5^ kBq/mL/min^3^, 2.0 × 10^2^ kBq/mL/min, 9.3 × 10^–1^ kBq/mL, and 4.5 kBq/mL, respectively) and decay parameters *b_1_* to *b_6_* (3.6, 2.1 × 10^1^, 2.1 × 10^1^, 2.5, 1.9 × 10^–2^, and 1.8 × 10^–1^ min^–1^, respectively) were derived from the average plasma AIF in the validation study (described below). Similarly, a theoretical whole-blood AIF [*C*_*b*_(*t*); Figure 2a] was generated using Equation (13) with the same gamma-distribution orders as *C*_*p*_(*t*), but with amplitude parameters *a_i_* (9.6 × 10^5^ kBq/mL/min^7^, 6.6 × 10^5^ kBq/mL/min^3^, 6.7 × 10^5^ kBq/mL/min^3^, 7.3 × 10^–1^ kBq/mL/min, 3.8 × 10^1^ kBq/mL, 1.3 kBq/mL) and decay parameters *b_i_* (1.5 × 10^1^, 2.3 × 10^1^, 2.3 × 10^1^, 1.8 × 10^–1^, 9.4 × 10^–1^, and 9.2 × 10^–3^ min^–1^) estimated from the mean measured blood AIF. A time offset (*t_0_*) of 0.5 min was used for both *C*_*p*_(*t*) and *C*_*b*_(*t*) to account for the typical delay between the start of the PET acquisition and the radiotracer injection time.

$$C_{p}\,\left(t\right)=\overset{6}{\underset{i=1}{\sum }}a_{i}\,{\left(t-t_{0}\right)}^{k_{i}}\,e^{-b_{i}\,\left(t-t_{0}\right)}$$ (13)

The reference TAC was generated using an influx rate constant $K_{1}^{\prime }$ = 0.05 mL/cm^3^/min and $k_{2}^{\prime }$ computed as $K_{1}^{\prime }/\alpha$, where α represents the average *K*_1_/*k*_2_ ratio. For [^11^C]CURB (60 min acquisition), α is approx. 2.7 (Rusjan et al., 2013), resulting in $k_{2}^{\prime }$∼ 0.0185 min^–1^. Simulated PET TACs were fit using Equation (10) while fixing $k_{2}^{\prime }$, resulting in two fitted parameters per TAC (i.e., *R_1_* and *k_f_*); all remaining micro- and macroparameters were computed *a posteriori*.

Since *R_1_* depends solely on *K_1_*, error in *R_1_* was evaluated by simulating tissue TACs with *K_1_* values ranging from 0.10 to 0.40 mL/cm^3^/min (step size of 0.001 mL/cm^3^/min), corresponding to *R_1_* values between 2 and 8. The efflux rate constant was computed as $k_{2}=R_{1}\,k_{2}^{\prime }$, and the irreversible trapping rate constant was fixed at *k_3_* = 0.055 min^–1^, corresponding to a λ*k*_3_ of approx. 0.15 mL/cm^3^/min. Blood volume (*V_b_*) was incorporated to each curve by generating PET TACs as *C*_*PET*_(*t*)=(1−*V*_*b*_)*C*_*T*_(*t*) + *V*_*b*_*C*_*b*_(*t*) (Figure 2b), with reference-region blood volume fixed at 0.02 mL/cm^3^ (consistent with the lower blood volume expected in WM), and tissue *V_b_* of 0.02, 0.05, and 0.10 mL/cm^3^.

Error in *R_i_* and *Rk*_3_ was assessed by simulating tissue TACs with *K_1_* values of 0.15, 0.25, and 0.35 mL/cm^3^/min, corresponding to *R_1_* values of 3, 5, and 7, respectively. For each TAC, *k_2_* computed as $k_{2}=R_{1}\,k_{2}^{\prime }$ (approx. 0.055, 0.093, and 0.130 min^–1^, respectively), while *k_3_* was varied from 0.018 to 0.093 min^–1^ in steps of 0.001 min^–1^. These parameter combinations resulted in theoretical λ*k*_3_ values ranging from 0.05 to 0.25 mL/cm^3^. Blood volume effects were again incorporated using tissue *V_b_* values of 0.02, 0.05, and 0.10 mL/cm^3^. Lastly, to further characterize the relationship between blood volume and macroparameter bias, simulations were repeated with *k_3_* fixed at 0.055 min^–1^ while varying *V_b_* continuously from 0.01 to 0.10 mL/cm^3^. Additional simulations can be found in Supplementary material.

### Validation of IRTM against AIF-based quantification

PET data acquired with [^11^C]CURB from healthy participants were used to validate the IRTM against AIF-based quantification. The initial study aimed at investigating brain FAAH levels at baseline and following acute nabilone exposure in healthy individuals (manuscript in preparation). All study procedures were approved by the Research Ethics Board (REB #098-2017) at the Centre for Addiction Mental Health (CAMH) and conducted in accordance with the Declaration of Helsinki ethical standards. All participants provided written informed consent for the use of their data in compliance with the Tri-Council Policy Statement of Ethical Conduct for Research Involving Humans.

Participants were recruited through community flyers, online advertisements, and ongoing studies. Participants completed a comprehensive screening interview, including a review of current and past medical, psychiatric, and substance use histories, state-level behavioral measures, and collection of urine and blood samples. Blood specimens underwent routine blood work and a comprehensive metabolic panel. Adult (ages 19–65) males and females were enrolled on the basis that they were willing and able to complete the study as per protocol and did not meet any of the exclusion criteria detailed below.

Urine toxicology was performed at screening to subsequently exclude participants with past 30-day use of drugs of abuse (including cannabis) and medications known to affect the central nervous system, mental and/or psychomotor functioning, or the study dosing regimen. Potential participants were excluded if they reported past or current serious unstable medical condition(s), neurological illness(s) or history of head trauma, blood coagulation history/disorder(s) or the use of anticoagulant medication, positive urine toxicology or clinically significant blood results, lifetime diagnosis of DSM-5 Axis 1 psychiatric disorders, lifetime substance abuse and/or dependence, current tobacco dependence, current suicidality or lifetime attempts, first-degree family history of psychotic disorders, known sensitivity to cannabinoids, or had any contraindications for PET or MRI.

#### Image acquisition

Dynamic PET data were acquired on a PET/computed tomography (CT) scanner [5-ring GE Discovery MI; 25-cm axial field-of-view (FoV)] in list mode for 60 min. Participants were scanned feet-first on a supine position, with head motion minimized using a fitted thermoplastic face mask (Tru-Scan Imaging, Annapolis, United States). Following a low-dose CT scan for attenuation correction, PET acquisition started approx. 30 s prior to radiotracer injection. List-mode PET data were binned into 22 time-frames (1 × background, 5 × 30 s, 1 × 45 s, 2 × 60 s, 1 × 90 s, 1 × 120 s, 1 × 210 s, and 10 × 300 s) and reconstructed using a filtered back-projection analytical algorithm. Dynamic PET images were reconstructed into a 256 × 256 × 89 matrix with voxel dimensions of 1.6 × 1.6 × 2.8 mm (FoV, 400 × 400 × 249 mm^3^). Corrections for decay, random coincidences, dead-time, detector normalization, attenuation, and scattering were applied. Magnetic resonance imaging (MRI) scans were collected on a 3-Tesla GE Discovery MR750 scanner using a 32-channel coil (model 3832016, Nova Medical Inc., United States). Anatomical images were acquired using a BRAVO sequence (3D inversion recovery-prepared fast spoiled gradient-recalled; repetition time, 6.768 ms; echo time, 3.016 ms; inversion time, 650 ms; flip angle, 8°; matrix, 256 × 256 × 200 voxels; FoV, 230 × 230 × 200 mm^3^; voxel size, 0.9 × 0.9 × 0.9 mm^3^).

#### Polymorphism genotyping

Since [^11^C]CURB binding affinity is influenced by a functional single-nucleotide polymorphism in the FAAH gene (rs324420 C > A; Pro129Thr) (Boileau et al., 2015b), all participants were genotyped for this variant. Genotyping was performed using a TaqMan SNP Genotyping assay (ID C_1897306_10; Life Technologies, Burlington, Canada) as per (Boileau et al., 2014).

#### Arterial blood data collection and analysis

Prior to PET scanning, a catheter was inserted into the radial artery by a certified respiratory therapist to enable arterial sampling. Continuous arterial blood activity was measured for the first 22 minutes of the scan using an automated blood sampling system (ABSS; PBS-101, Comecer S.p.A., Italy) to capture the radiotracer first pass peak. Manual arterial blood samples ranging from 5 to 10 mL were collected at predefined time points relative to injection time (i.e., − 5, 3, 7, 12, 15, 20, 30, 45, and 60 min). Post-injection arterial samples were used to determine the blood-to-plasma ratio (BPR) and perform radiometabolite analysis with high-performance liquid chromatography, from which the parent radiotracer in plasma fraction (PF) was determined.

AIFs were generated using an in-house MATLAB-based graphical user interface (Narciso et al., 2024b). Briefly, the continuous ABSS whole-blood curve (511 keV energy window) was fit with four gamma distribution functions (typically orders 9, 1, 1, and 0), and the fitted curve was used to replace data points corresponding to manual sampling intervals. The ABSS curve was subsequently scaled to discrete whole-blood activity concentration measurements from manual samples. Radiometabolite correction was performed by fitting the BPR measurements to a bi-exponential function and PF with a Hill function. The resulting AIF was corrected for delay and dispersion (integrals method; dispersion constant of 13 s; Brender et al., 2025), and denoised with the MATLAB function *smoothdata* [Savitzky-Golay filter (sgolay); window of 10 data points].

#### Imaging data processing

Dynamic PET images were corrected for inter-frame motion by rigidly realigning each time frame to a reference frame with PMOD (version 4.5; PMOD Technologies LLC, Bruker, Zurich, Switzerland). The corresponding anatomical MRI was used for automated delineation of VOIs using in-house MATLAB (R2024b, The MathWorks, Inc.) scripts based on statistical parametric mapping (version 12, SPM12).^1^ Each subject’s MRI was co-registered to the motion-corrected dynamic PET image, segmented into tissue probability maps, and normalized to the Montreal Neurological Institute (MNI; McGill University, Canada) space. Target GM VOIs were defined using the Hammers atlas (Hammers et al., 2003) and included the frontal, temporal, parietal, and occipital lobes, anterior and posterior cingulate cortices, hippocampus, amygdala, basal ganglia, thalamus, and insula. The resulting 11-VOI mask was transformed from MNI space back into native PET space using the inverse normalization parameters and refined based on individual tissue segmentation to include only GM voxels. TACs were extracted from the dynamic image as the average activity concentration across all voxels within each VOI and used for subsequent kinetic analysis.

#### Kinetic modeling with AIF

Each VOI TAC was fit to the i2TCM with the radiometabolite-corrected AIF to estimate the composite parameter λ*k*_3_ (λ = *K*_1_/*k*_2_) and the net influx rate constant *K_i_* ( = *K*_1_*k*_3_/*k*_*f*_). Model fitting was performed using a weighted non-linear least-squares approach that implemented the MATLAB optimization function *lsqnonlin*. Three kinetic parameters were estimated (i.e., *K_1_*, *k_2_*, and *k_3_*). Initial parameter values were 0.25 mL/cm^3^/min, 0.10 min^–1^, and 0.05 min^–1^ for *K_1_*, *k_2_*, and *k_3_*, respectively (equivalent to λ*k*_3_ = 0.125 mL/cm^3^). Respective upper bounds were set to 1 mL/cm^3^/min, 1 min^–1^, and 1 min^–1^, while all lower bounds were set to zero. The cost function was defined as a weighted residual sum of squares (ϕ, see Equation 14, for *n* = 1) with weights defined according to Equation (15). The fractional blood volume parameter (*V_b_*) was fixed to 0.05 mL/cm^3^ for GM regions.

Similarly, WM cluster TACs (extracted as described in Implementation of IRTM at VOI- and voxel-level) were fit separately to the 1TCM with the metabolite-corrected AIF, under the assumption of negligible specific binding. Two kinetic parameters were estimated (i.e., $K_{1}^{\prime }$ and $k_{2}^{\prime }$) using weighted non-linear least-squares optimization [*lsqnonlin*; ϕ defined by Equation (14) for *n* = 1]. Initial values were 0.25 mL/cm^3^/min and 0.10 min^–1^ for $K_{1}^{\prime }$ and $k_{2}^{\prime }$, respectively, with upper bounds of 1 mL/cm^3^/min and 1 min^–1^ and lower bounds set to zero. The *V_b_* parameter was fixed to 0.02 mL/cm^3^.

#### Clustering of WM TACs

**FIGURE 3:**
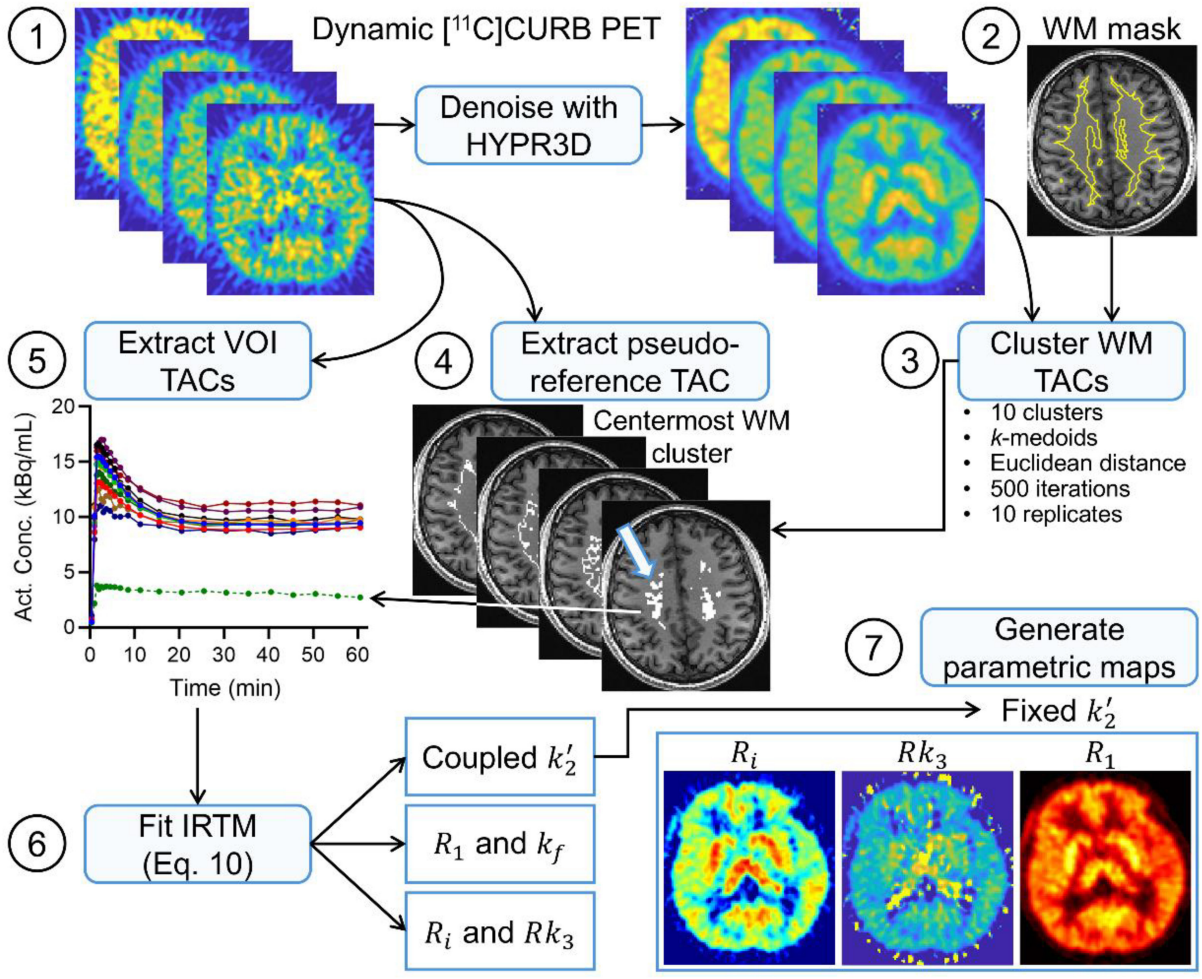
Overview of the IRTM implementation for validation with human [^11^C]CURB PET data. (1) The PET image is denoised using HYPR3D exclusively for WM clustering. (2) Using an eroded WM mask, (3) WM voxel TACs are grouped based on kinetic similarity using a partition-based clustering algorithm, and (4) the centermost WM cluster is selected as a pseudo-reference region. (5) VOI TACs are extracted from the dynamic PET image and (6) VOI and WM reference TACs are entered into the IRTM fitting routine to estimate VOI-specific parameters and the common *k*2′. Finally, (7) the estimated coupled-*k*2′ is used to generate voxel-wise parametric maps.

The reference region was obtained from WM voxels using a data-driven clustering approach based on functional similarity (Figure 3). For clustering only, dynamic PET images were denoised by applying the 3D highly constrained backprojection (HYPR3D) method (Christian et al., 2010), using a Gaussian filter kernel of standard deviation 3 voxels (corresponding to 4.8 × 4.8 × 8.3 mm^3^) to improve the signal-to-noise ratio.

To ensure high tissue purity, each participant’s WM tissue probability map was thresholded at 80% and eroded using a spherical structuring element with a radius of 1 voxel. Small groups of 3 or fewer voxels were removed from each slice to reduce isolated noise. This conservative masking strategy ensured that included WM voxels were approx. 3 mm away from adjacent structures, thereby reducing signal spill-in from GM and spill-out into cerebrospinal fluid voxels.

Using this refined WM mask, TACs were clustered using a partition-based clustering algorithm (Euclidean distance, 500 iterations, 10 replicates, 10 clusters), implemented using the *k*-medoids MATLAB function *kmedoids*. The resulting functional atlas was then used to extract the 10 WM cluster TACs from the original (non-HYPR3D) dynamic PET image. Then, the centermost WM cluster—representing the core WM region most isolated from GM contamination—was selected as reference region for IRTM modeling. This target cluster was identified by visually selecting the cluster located deepest within the spatial center of the WM mask (i.e., maximizing distance from GM and cerebrospinal fluid boundaries) and confirmed by verifying that its corresponding TAC exhibited the lowest area-under-the-curve among all generated clusters.

The anatomical localization of each cluster was determined by mapping the cluster volumes to the stereotaxic probabilistic WM atlas developed by the International Consortium of Brain Mapping (ICBM) using diffusion tensor imaging (DTI) from 81 participants (ICBM-DTI-81) (Mori et al., 2008; Oishi et al., 2008).

#### Implementation of IRTM at VOI- and voxel-level

Quantification of FAAH activity using IRTM was performed by fitting each VOI TAC to Equation (10) using a coupled-fit approach implemented in MATLAB via the *lsqnonlin* optimization routine. The fitting procedure included a total of *2n+1* parameters, where *n* is the number of VOIs. These consisted of VOI-specific parameters (*R_1_* and *k_f_*) and a single shared reference efflux rate constant ($k_{2}^{\prime }$). Initial parameter values for *R_1_* and *k_f_* were set to 3 and 0.15 min^–1^, respectively, with upper bounds of 10 and 1 min^–1^ and lower bounds set to zero. The initial value for $k_{2}^{\prime }$ was 0.015 min^–1^, with bounds of 0 to 1 min^–1^. The residual objective function (ϕ) was defined as a weighted residual sum of squares given by Equation (14).


$$\phi =\overset{n}{\underset{j=1}{\sum }}\overset{T}{\underset{i=1}{\sum }}w_{i}\,{\left(C_{j}^{f\,i\,t}\,\left(t_{i}\right)-C_{j}\,\left(t_{i}\right)\right)}^{2}$$ (14)

where *C*_*j*_(*t*) denotes the measured activity concentration of the *j^th^* VOI, $C_{j}^{f\,i\,t}\,\left(t\right)$ the corresponding model estimate (i.e., Equation 10), and *T* the total number of time frames. Weights (*w_i_*) for the fitting routine were defined as the inverse of variance of the PET measurement error and included a decay component (Equation 15).


$$w_{i}=\frac{\Delta \,t_{i}\,e^{-\frac{\ln\,\left(2\right)}{20.38}\,t_{i}}}{\bar{C_{P\,E\,T}}\,\left(t_{i}\right)}$$ (15)

where Δ*t*_*i*_ is the frame duration, *t_i_* the mid-frame time, and $\bar{C_{P\,E\,T}}\,\left(t\right)$ the average TAC across GM VOIs. Weights were normalized to the maximum weight value and those corresponding to low activity concentration time-frames were removed (i.e., < 10% of maximum $\bar{C_{P\,E\,T}}\,\left(t\right)$ value).

The resulting 11 GM VOI TACs and the WM reference TAC were used as input to the coupled-fit algorithm to estimate VOI-specific parameters and the shared $k_{2}^{\prime }$. Finally, the resulting $k_{2}^{\prime }$ was subsequently used to generate voxel-wise parametric maps of *R_1_*, *k_f_*, *R_i_*, and *Rk*_3_.

### Statistics

Standardized uptake values (SUVs) were computed as the ratio between activity concentration (in kBq/mL) and the injected activity (MBq) normalized by body weight (kg). The model selection criterion (MSC) was used to compare i2TCM and 1TCM fits of WM cluster TACs, computed as $M\,S\,C=\ln\,\left[\sum_{i=1}^{T}w_{i}\,{\left(y\,\left(t_{i}\right)-\bar{y}\right)}^{2}/\sum_{i=1}^{T}w_{i}\,{\left(y\,\left(t_{i}\right)-\hat{y}\,\left(t_{i}\right)\right)}^{2}\right]-2\,p/T$, where *y*(*t*) represents the PET TAC, $\hat{y}\,\left(t\right)$ the model fit, $\bar{y}=\sum_{i=1}^{T}w_{i}\,y\,\left(t_{i}\right)/\sum_{i=1}^{T}w_{i}$, and *p* the number of fitted parameters. Relationship between IRTM and i2TCM was evaluated by means of the mixed-effects linear regression, defined as *y*_*s*,*j*_ = (β_0_ + *b*_0,*s*_) + (β_1_ + *b*_1,*s*_)*x*_*s*,*j*_ + ε_*s*,*j*_, where β_0_ and β_1_ are the population-level intercept and slope, respectively; *b*_0,*s*_ and *b*_1,*s*_ are subject-specific random effects for the intercept and slope, respectively; ε_*s*,*j*_ is the residual error; and the subscripts *s* and *j* represent each subject and VOI, respectively. The mixed-effects linear regression is presented with its 95% confidence interval. The relationship between AIF- and IRTM-derived estimates was assessed using a linear mixed-effects model using IRTM measurement, genotype, and their interaction as fixed effects. VOI was included as a fixed-effect covariate and a random intercept for each subject was used. Significance was determined using type III ANOVA marginal tests. Correlation was assessed by means of the Pearson correlation coefficient (*r*). Statistical tests were performed using GraphPad Prism (version 10, San Diego, United States). Statistical significance was defined by a level of 0.05. Measurements are presented in terms of mean ± one standard deviation alongside their percent standard error (SE) in square brackets when relevant.

## Results

### Simulations

**FIGURE 4:**
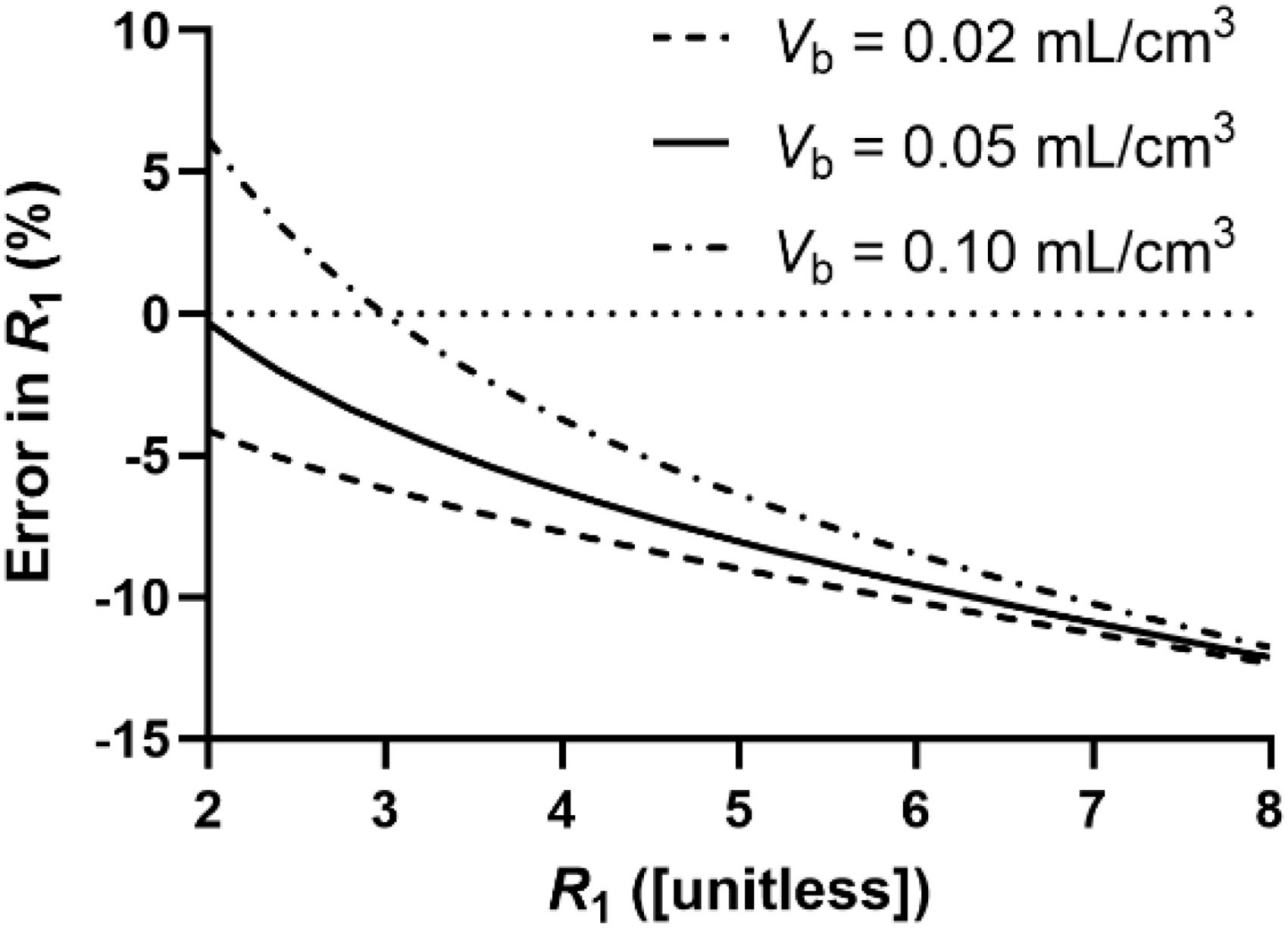
Predicted error (%) in *R*_1_ as a function of theoretical *R*_1_ for blood volumes fractions of 0.02 (black dashed line), 0.05 (solid), and 0.10 mL/cm^3^ (dot-dashed). Reference region blood volume was fixed at 0.02 mL/cm^3^. The reference TAC was generated using *K*1′ = 0.05 mL/cm^3^/min and *k*2′ = 0.0185 min^–1^. Target-region microparameters were defined as *k*_2_ = *R*_1_*k*2′, and *k*_3_ of 0.055 min^–1^, corresponding to a λ*k*_3_ of approx. 0.15 mL/cm^3^/min.

The predicted error in *R_1_* increased monotonically with increasing simulated *R_1_*, resulting in progressively greater underestimation at higher *R_1_* (Figure 4). Notably, the influence of blood volume contamination on *R_1_* estimation was reduced at higher values of *K_1_*, indicating lower sensitivity of *R_1_* bias to vascular contribution under conditions of higher radiotracer delivery.

**FIGURE 5:**
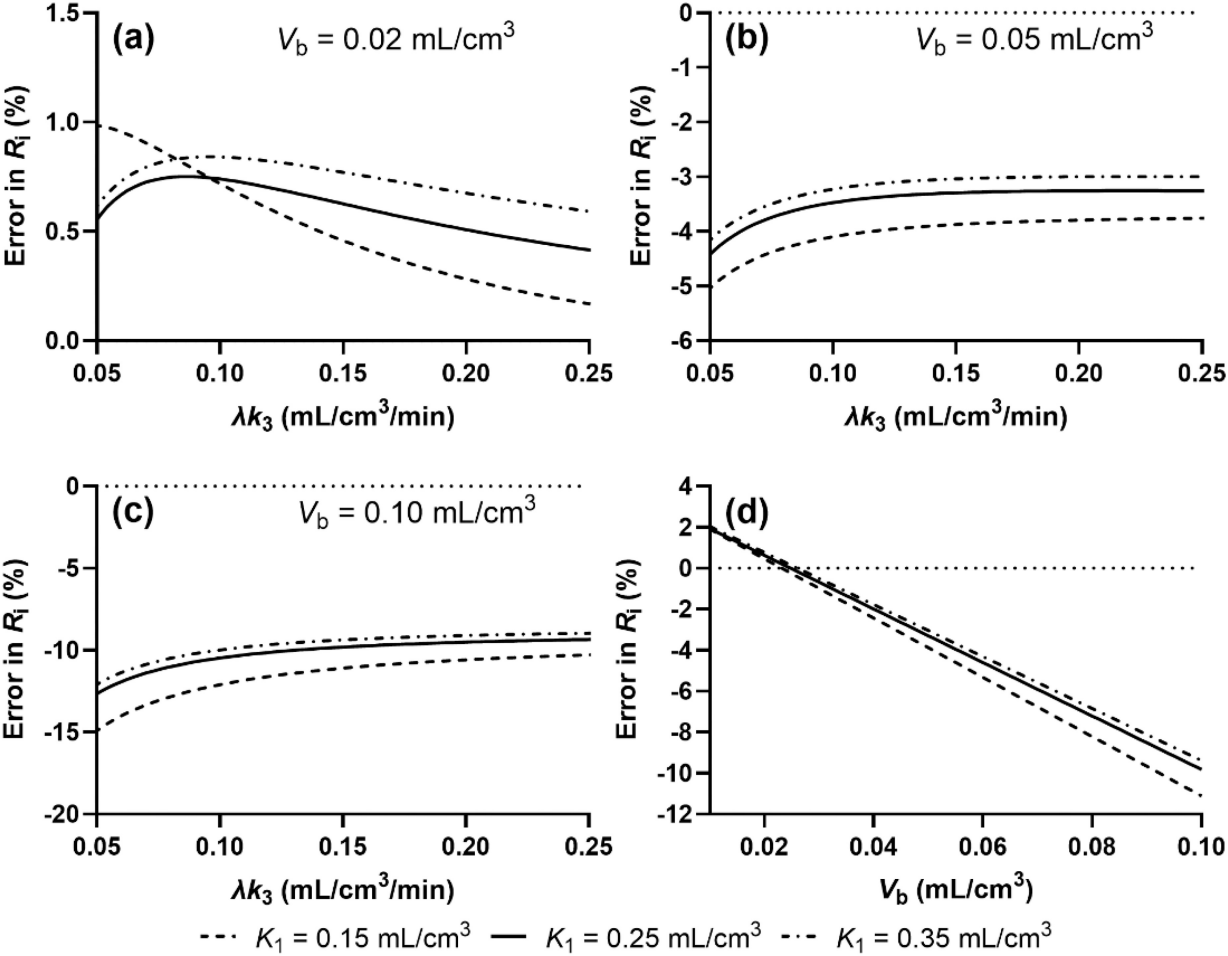
Predicted error (%) in *R*_*i*_ as a function of theoretical λ*k*_3_ for *K*_1_ values of 0.15 (black dashed line), 0.25 (solid), and 0.35 mL/cm^3^/min (dot-dashed). Corresponding *k*_2_ values were approx. 0.055, 0.093, and 0.130 min^–1^ ($k_{2}=R_{1}\,k_{2}^{{}^{\prime }}$). The reference TAC was generated with $K_{1}^{{}^{\prime }}$ = 0.05 mL/cm^3^/min and $k_{2}^{{}^{\prime }}$ = 0.0185 min^–1^, with blood volume fixed at 0.02 mL/cm^3^. Simulations were performed for **(a)** *V*_*b*_ = 0.02 mL/cm^3^, **(b)** *V*_*b*_ = 0.05 mL/cm^3^, and **(c)** *V*_*b*_ = 0.10 mL/cm^3^, with *k*_3_ ranging from 0.018 to 0.093 min^–1^. **(d)** Simulations were repeated with *V*_*b*_ varying from 0.01 to 0.10 mL/cm^3^ while fixing *k*_3_ to 0.055 min^–1^ (λ*k*_3_ = 0.15 mL/cm^3^/min).

Simulations demonstrated that underestimation of *R_i_* increases with increasing blood volume fraction (Figure 5). However, for a given *V_b_* value, the predicted error in *R_i_* remained relatively constant across the range of λ*k*_3_ values simulated in this study. This suggests that *R_i_* bias is primarily driven by vascular contamination rather than by degree of irreversible binding.

**FIGURE 6:**
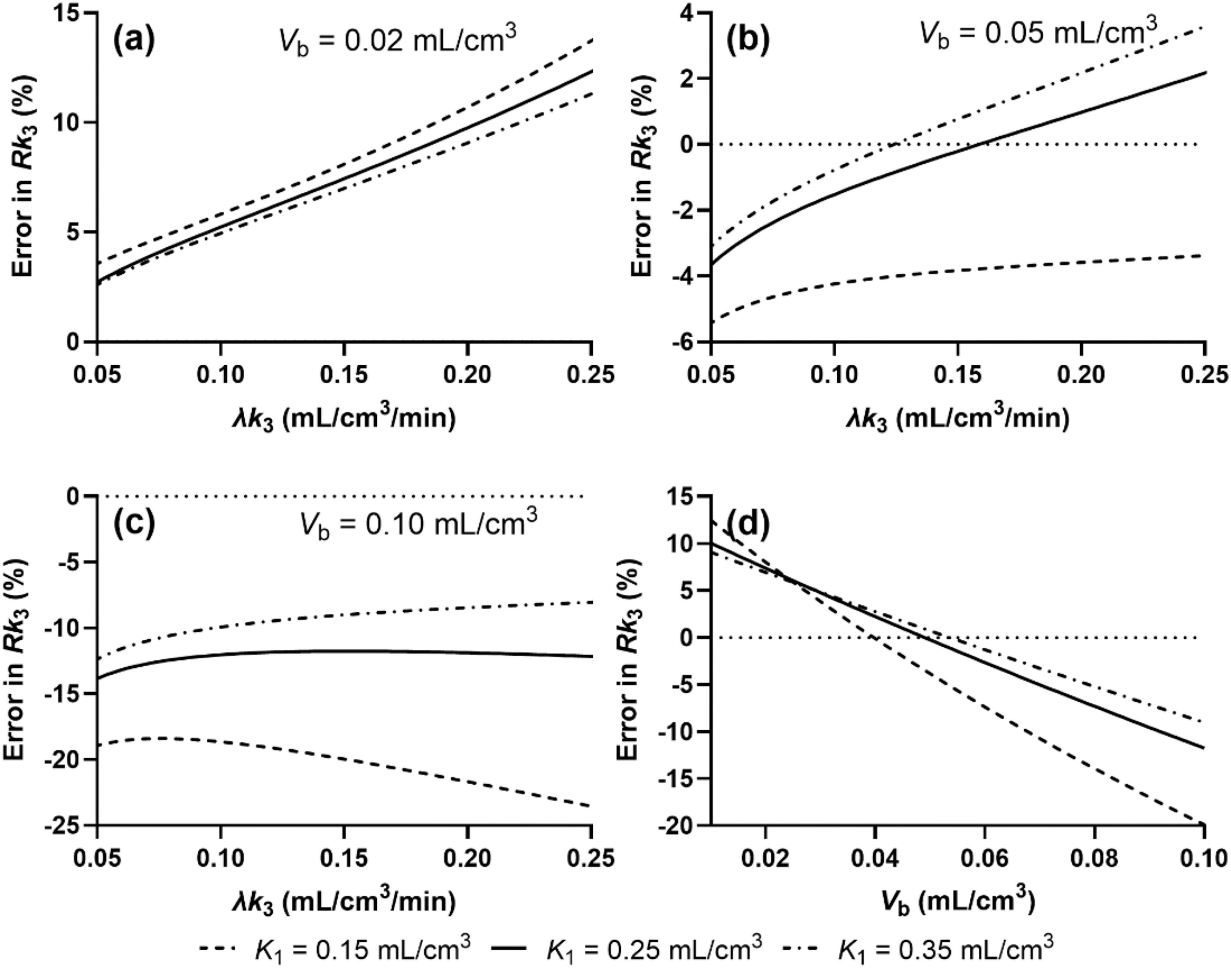
Predicted error (%) in *Rk*_3_ as a function of theoretical λ*k*_3_ for *K*_1_ values of 0.15 (black dashed line), 0.25 (solid), and 0.35 mL/cm^3^/min (dot-dashed). Corresponding *k*_2_ values were approx. 0.055, 0.093, and 0.130 min^–1^ ($k_{2}=R_{1}\,k_{2}^{{}^{\prime }}$). The reference TAC was generated with $K_{1}^{{}^{\prime }}$ = 0.05 mL/cm^3^/min and $k_{2}^{{}^{\prime }}$ = 0.0185 min^–1^, with blood volume fixed at 0.02 mL/cm^3^. Simulations were performed for **(a)** *V*_*b*_ = 0.02 mL/cm^3^, **(b)** *V*_*b*_ = 0.05 mL/cm^3^, and **(c)** *V*_*b*_ = 0.10 mL/cm^3^, with *k*_3_ ranging from 0.018 to 0.093 min^–1^. **(d)** Simulations were repeated with *V*_*b*_ varying from 0.01 to 0.10 mL/cm^3^ while fixing *k*_3_ to 0.055 min^–1^ (λ*k*_3_ = 0.15 mL/cm^3^/min).

In contrast, the predicted error in *Rk*_3_ showed a more complex dependency, with estimation accuracy influenced by both λ*k*_3_ and blood volume fraction levels (Figure 6). Specifically, higher blood volume fractions exacerbated bias in *Rk*_3_, while the magnitude and direction of error varied as a function of λ*k*_3_, suggesting an interaction between *Rk*_3_, irreversible trapping, and vascular signal contamination.

### Centermost WM cluster as reference region

PET [^11^C]CURB data from 10 healthy participants (31 ± 9 years; 73 ± 10 kg; 4 females and 6 males; 2 AA, 3 AC, and 5 CC FAAH genotypes; average injected activity, 390 ± 30 MBq [range 330–440 MBq]) acquired at baseline were included in this study.

**FIGURE 7:**
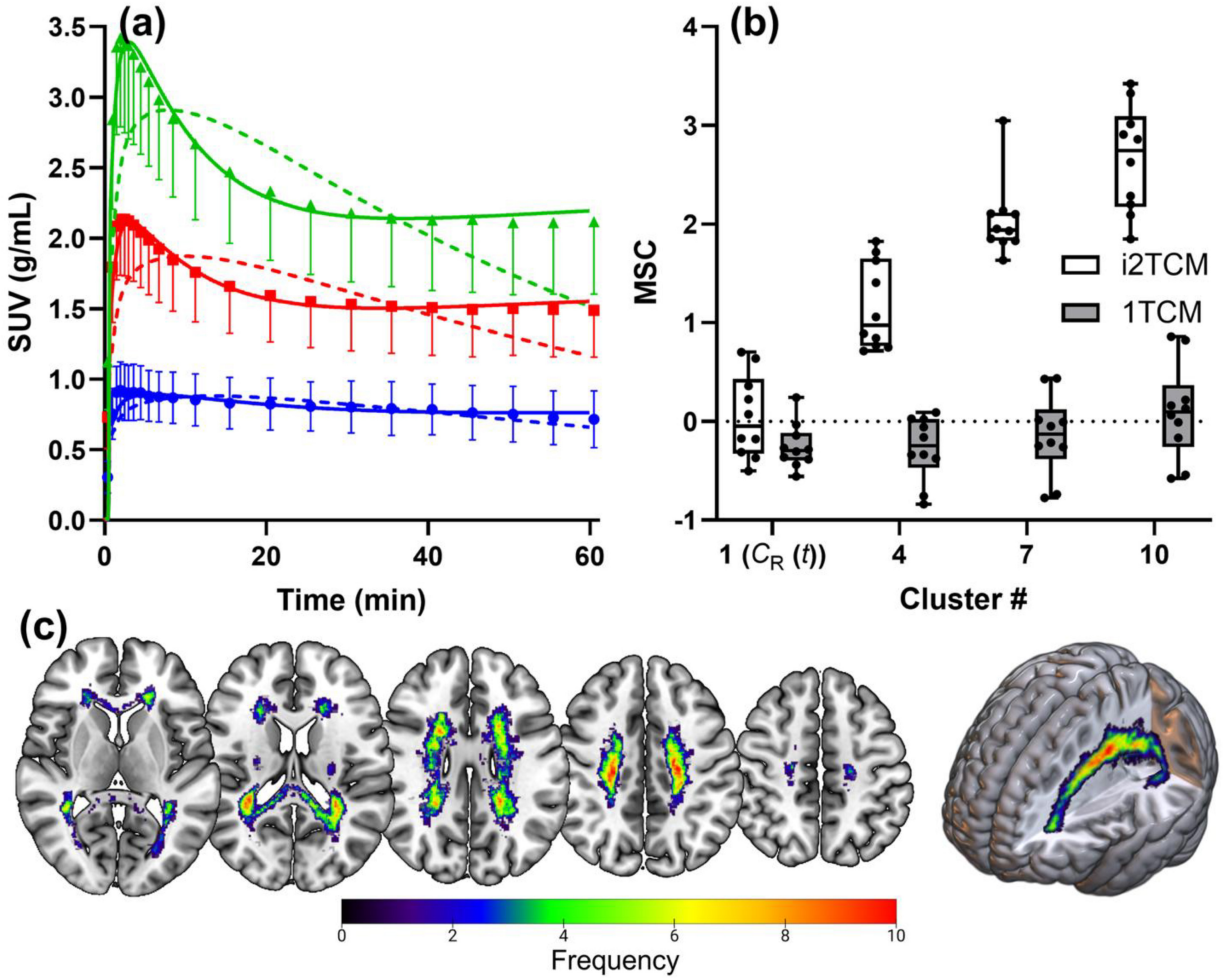
**(a)** Representative cluster TACs (*n* = 10) expressed as SUV (in g/mL). The green curve (triangles; cluster #10), obtained from the cluster located closest to deep GM structures and in the brainstem, exhibits the largest discrepancy between i2TCM (solid curves) and 1TCM (dashed curves) fits. The blue curve (circles; cluster #1), corresponding to the centermost WM cluster, was selected as the reference TAC in this study (*C*_*R*_(*t*)), and shows closer congruency between the two models. The red curve (squares; cluster #7) represents an intermediate cluster situated between these two extremes. **(b)** MSC values (*n* = 10) demonstrating convergence between i2TCM and 1TCM fits for clusters located progressively closer to the center of the WM tissue. **(c)** Frequency map showing the voxels selected as part of the centermost WM cluster (i.e., the reference region). Note, the anatomical MRI shown is a template used for visualization purposes only; individual participant anatomy and precise voxel-wise spatial fidelity may differ from this representative image.

WM cluster TACs exhibited increasing concordance between i2TCM and 1TCM fits as clusters were located progressively closer to the center of WM tissue (Figure 7a), as reflected by the corresponding MSC values (Figure 7b). Notably, the cluster selected as the reference TAC in this study (Figure 7c) demonstrated closer congruency between the two models, resulting in average 1TCM-derived $K_{1}^{\prime }$ and $k_{2}^{\prime }$ of 0.051 ± 0.007 mL/cm^3^/min and 0.0128 ± 0.0030 min^–1^, respectively. Average volume of the centermost WM cluster (i.e., reference region) was 15.1 ± 3.0 mL. Across the cohort, the reference WM cluster was primarily distributed within the corona radiata and corpus callosum, with additional voxels identified in the thalamic radiation, superior longitudinal fasciculus, tapetum, and internal capsule. Details for all WM clusters are also shown in Supplementary Figure 1.

### Validation of IRTM against AIF-based quantification

**FIGURE 8:**
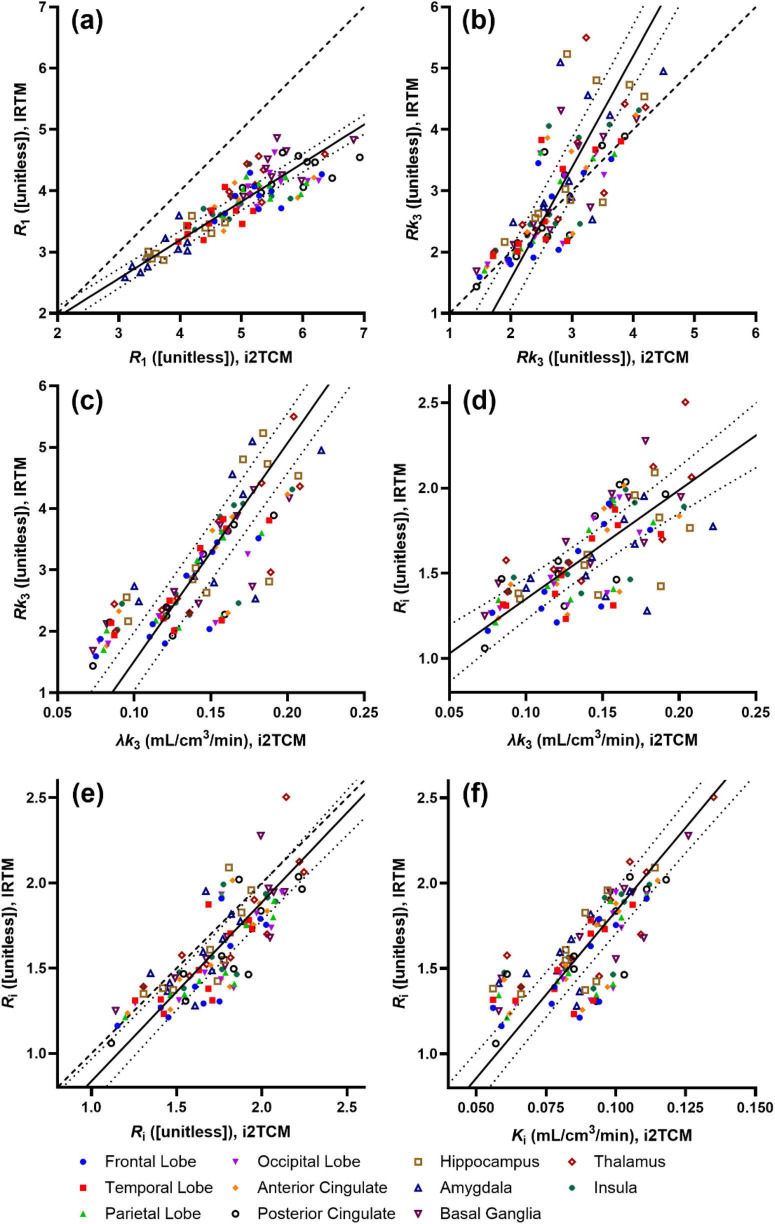
**(a)** Linear regression comparing i2TCM-derived and IRTM-derived *R*_1_ estimates (mixed-effects linear regression: *y* = 0.63*x* + 0.68, *R*^2^ = 0.95). **(b,c)** Comparison of IRTM-derived *Rk*_3_ with i2TCM measurements of (b) *Rk*_3_ (*y* = 1.81*x*−2.07, *R*^2^ = 0.98) and **(c)** λ*k*_3_ (*y* = 35.6*x*−2.04, *R*^2^ = 0.98). **(d–f)** Comparison of IRTM-derived *R*_*i*_ with i2TCM-derived **(d)** λ*k*_3_ (*y* = 6.38*x* + 0.71, *R*^2^ = 0.90), **(e)** *R*_*i*_ (*y* = 1.05*x*−0.22, *R*^2^ = 0.96), and **(f)** *K*_*i*_ (*y* = 19.6*x*−0.12, *R*^2^ = 0.96). Black solid lines represent mixed-effects linear regression fits, with dotted lines indicating the ± 95% confidence intervals.

IRTM- and i2TCM-derived estimates are summarized in Table 1 and Supplementary Table 1. Using the centermost WM cluster as pseudo reference region within the IRTM framework resulted in coupled-fit $k_{2}^{\prime }$ of 0.010 ± 0.002 min^–1^ (standard error = 2.9 ± 0.6%). Good predictive association between i2TCM- and IRTM-derived *R_1_* estimates was observed (Figure 8a), albeit with a clear *K_1_*-dependent bias, as predicted by the simulations study (Figure 4). Mixed-effects linear regression yielded *y* = 0.63*x* + 0.68 (*R*^2^ = 0.95). Linear regression performed separately for each VOI resulted in mean slope of 0.38 ± 0.12, a mean intercept of 2.0 ± 0.6, and a mean *R*^2^ of 0.42 ± 0.17. Similarly, correlation analysis at the VOI level yielded a mean *r* of 0.64 ± 0.12, with statistically significant correlations observed for the anterior cingulate, hippocampus, amygdala, and basal ganglia.

**TABLE 1:** Summary of results obtained with i2TCM and IRTM.

VOI	*R*_1_ ([unitless]) [%SE]		λ*k*_3_ (mL/cm^3^/min) [%SE]	*Rk*_3_ ([unitless]) [%SE]		*K*_*i*_ (mL/cm^3^/min) [%SE]	*R*_*i*_ ([unitless]) [%SE]	
	i2TCM	IRTM	i2TCM	i2TCM	IRTM	i2TCM	i2TCM	IRTM
Frontal lobe	5.26 ± 0.50	3.90 ± 0.27 [1.7 ± 0.4]	0.127 ± 0.034 [4.8 ± 2.1]	2.49 ± 0.62	2.45 ± 0.75 [5.8 ± 1.2]	0.085 ± 0.018 [3.4 ± 1.6]	1.66 ± 0.27	1.47 ± 0.27 [3.3 ± 0.7]
Temporal lobe	4.54 ± 0.41	3.51 ± 0.27 [1.8 ± 0.4]	0.135 ± 0.033 [5.3 ± 2.2]	2.65 ± 0.62	2.76 ± 0.80 [6.6 ± 1.3]	0.084 ± 0.015 [3.4 ± 1.5]	1.65 ± 0.23	1.51 ± 0.24 [3.4 ± 0.7]
Parietal lobe	5.37 ± 0.45	4.00 ± 0.20 [1.7 ± 0.4]	0.132 ± 0.034 [4.7 ± 2.1]	2.60 ± 0.62	2.64 ± 0.75 [5.5 ± 1.0]	0.088 ± 0.018 [3.3 ± 1.6]	1.73 ± 0.27	1.56 ± 0.26 [3.1 ± 0.6]
Occipital lobe	5.51 ± 0.42	4.10 ± 0.27 [1.7 ± 0.4]	0.131 ± 0.033 [4.7 ± 1.8]	2.58 ± 0.60	2.59 ± 0.74 [5.3 ± 0.9]	0.088 ± 0.018 [3.3 ± 1.4]	1.73 ± 0.28	1.56 ± 0.27 [3.0 ± 0.6]
Anterior cingulate	5.03 ± 0.66	3.76 ± 0.32 [1.7 ± 0.4]	0.137 ± 0.036 [4.0 ± 1.7]	2.71 ± 0.67	2.83 ± 0.87 [6.0 ± 1.0]	0.088 ± 0.017 [2.6 ± 1.2]	1.73 ± 0.26	1.58 ± 0.27 [3.1 ± 0.6]
Posterior cingulate	5.91 ± 0.56	4.31 ± 0.25 [1.6 ± 0.4]	0.135 ± 0.037 [3.9 ± 1.7]	2.65 ± 0.70	2.70 ± 0.86 [5.0 ± 1.0]	0.092 ± 0.020 [2.8 ± 1.4]	1.80 ± 0.34	1.62 ± 0.33 [2.9 ± 0.7]
Hippocampus	3.97 ± 0.47	3.18 ± 0.28 [1.9 ± 0.4]	0.155 ± 0.039 [6.4 ± 2.4]	3.05 ± 0.71	3.53 ± 1.15 [7.5 ± 1.5]	0.086 ± 0.016 [3.5 ± 1.5]	1.70 ± 0.22	1.63 ± 0.27 [3.1 ± 0.6]
Amygdala	3.65 ± 0.38	2.98 ± 0.30 [1.9 ± 0.4]	0.155 ± 0.037 [6.2 ± 2.4]	3.06 ± 0.68	3.54 ± 1.04 [8.4 ± 1.9]	0.083 ± 0.013 [3.2 ± 1.3]	1.64 ± 0.17	1.58 ± 0.22 [3.3 ± 0.7]
Basal Ganglia	5.56 ± 0.60	4.34 ± 0.39 [1.6 ± 0.3]	0.142 ± 0.042 [5.2 ± 2.2]	2.79 ± 0.75	3.01 ± 0.93 [4.9 ± 1.0]	0.093 ± 0.022 [3.6 ± 1.8]	1.82 ± 0.31	1.73 ± 0.31 [2.6 ± 0.5]
Thalamus	5.27 ± 0.43	4.18 ± 0.29 [1.6 ± 0.4]	0.150 ± 0.045 [4.9 ± 2.0]	2.93 ± 0.79	3.27 ± 1.18 [5.2 ± 1.1]	0.095 ± 0.022 [3.3 ± 1.5]	1.85 ± 0.32	1.78 ± 0.36 [2.6 ± 0.6]
Insula	4.89 ± 0.49	3.78 ± 0.29 [1.7 ± 0.4]	0.143 ± 0.036 [4.6 ± 1.6]	2.83 ± 0.68	3.06 ± 0.90 [5.9 ± 1.0]	0.090 ± 0.017 [2.9 ± 1.1]	1.76 ± 0.25	1.65 ± 0.25 [2.9 ± 0.5]
WM (reference region)	1.16 ± 0.07	–	0.060 ± 0.022 [6.1 ± 4.5]	1.18 ± 0.42	–	0.029 ± 0.007 [3.5 ± 3.0]	0.57 ± 0.13	–

Results are expressed in terms of mean ± one standard deviation, alongside the percent standard error (%SE) when applicable.

The *Rk*_3_ macroparameter exhibited substantial between-subject variability (Figure 8b; *y* = 1.81*x*−2.06, *R*^2^ = 0.98). Despite this variability, IRTM-derived *Rk*_3_ estimates showed good linear relationship with i2TCM-derived values at the VOI level (slope = 1.00 ± 0.07, intercept = 0.19 ± 0.19, *R*^2^ = 0.57 ± 0.09, *r* = 0.75 ± 0.06), with statistically significant correlations observed for all VOIs except the amygdala. Comparable results were observed when comparing IRTM-derived *Rk*_3_ with i2TCM-derived λ*k*_3_ (Figure 8c; *y* = 35.6*x*−2.04, *R*^2^ = 0.98). VOI-level analysis yielded a mean slope of 19.6 ± 1.4 min cm^3^/mL, a mean intercept of 0.19 ± 0.13, a mean *R*^2^ of 0.64 ± 0.07, and a mean *r* of 0.80 ± 0.04, with significant correlations observed across all VOIs. The λ*k*_3_-dependence of *Rk*_3_ bias predicted by our simulations is clear in Figures 8b,c.

*R_i_* estimates obtained with IRTM showed reduced subject-specific bias and demonstrated strong linear relationship with i2TCM-derived λ*k*_3_ (Figure 8d; *y* = 6.38*x* + 0.71, *R*^2^ = 0.90). VOI-level analysis yielded a mean slope of 5.5 ± 1.2 min cm^3^/mL, a mean intercept of 0.84 ± 0.14, a mean *R*^2^ of 0.54 ± 0.13, and a mean *r* of 0.72 ± 0.10, with significant correlations observed for all VOIs except the amygdala. Similarly, there was strong association between IRTM-derived *R_i_* and i2TCM-derived estimates of *R_i_* (Figure 8e; *y* = 1.05*x*−0.21, *R*^2^ = 0.96; per VOI: slope = 0.83 ± 0.06, intercept = 0.17 ± 0.10, *R*^2^ = 0.63 ± 0.09, *r* = 0.79 ± 0.06; significance observed across all VOIs) and *K_i_* (Figure 8f; *y* = 19.6*x*−0.12, *R*^2^ = 0.96; per VOI: slope = 11.9 ± 0.9 min cm^3^/mL, intercept = 0.56 ± 0.09, *R*^2^ = 0.58 ± 0.09, *r* = 0.76 ± 0.06; significance observed across all VOIs).

**FIGURE 9:**
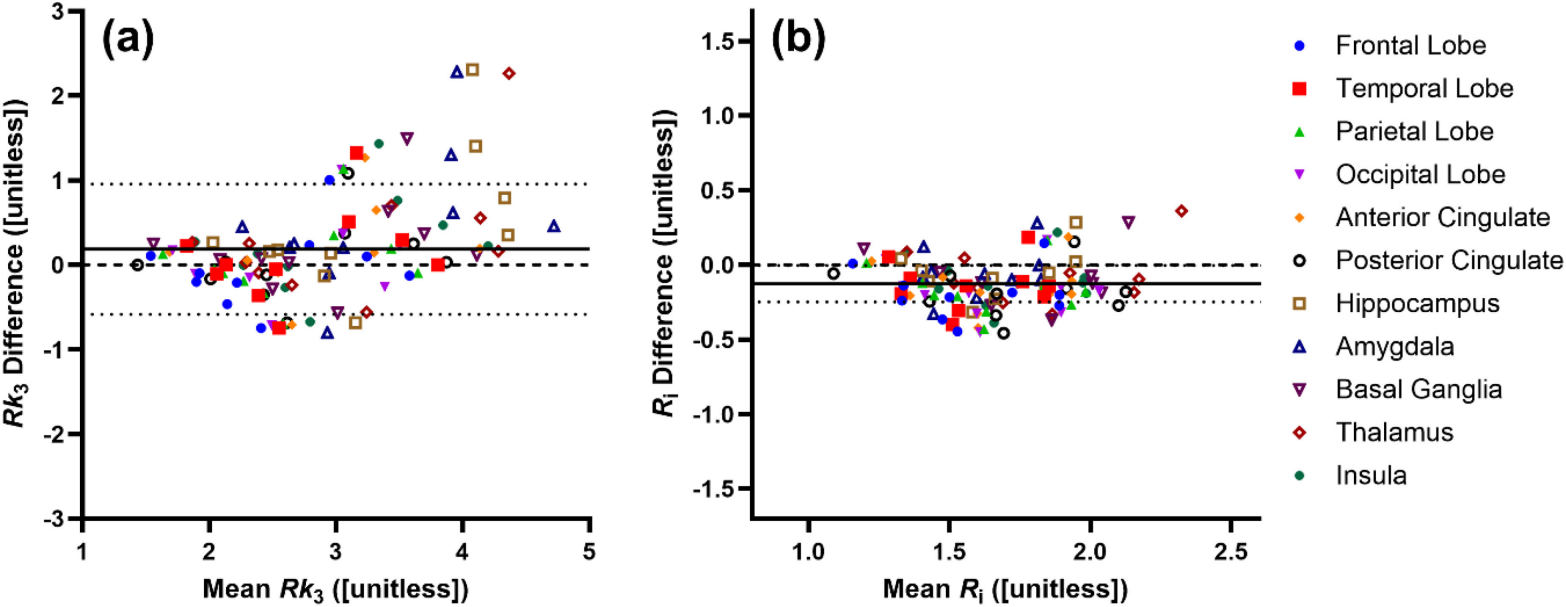
Bland-Altman plots showing the bias in macroparameters estimated with IRTM relative to AIF-based estimates. **(a)** *Rk*_3_ difference plotted against the mean *Rk*_3_. **(b)** *R*_*i*_ difference plotted against the mean *R*_*i*_. The dashed black line indicates the mean bias, while the dotted black lines represent the 95% limits of agreement. The *y*-axis scale in **(b)** was selected to facilitate comparison with the magnitude of *Rk*_3_ differences observed in **(a)**.

Bland-Altman analysis of *Rk*_3_ and *R_i_* is shown in Figure 9. Errors in *Rk*_3_ estimates ranged from −1.4% (± 18.6%) for the frontal lobe to 16.8% (± 27.9%) for the amygdala. Corresponding errors in IRTM-derived *R_i_* values ranged from −11.2% (± 9.9%) for the frontal lobe to −3.1% ( ±10.4%) for the amygdala. Although slightly biased, *R_i_* estimates obtained with IRTM presented higher precision (i.e., lower variability) and better agreement compared to *Rk*_3_ measurements. Importantly, the mean bias observed in *R_i_* across VOIs (approx. −7%) was consistent with predictions from our simulation study. This systematic bias in *R_i_* was eliminated when IRTM was implemented with an explicit blood volume correction (Supplementary Equation 1, Supplementary Figure 2, and Supplementary Table 2), confirming that vascular contamination is the primary source of the observed discrepancy.

**FIGURE 10:**
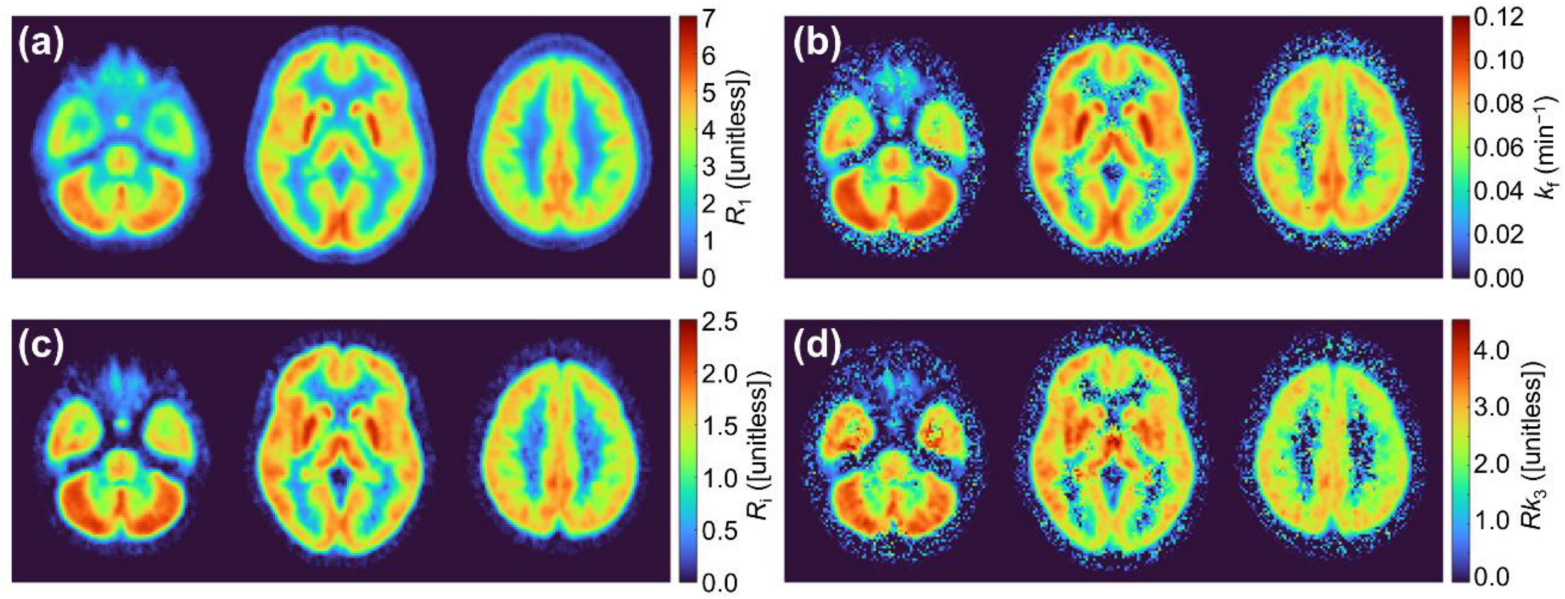
Group-average parametric maps (*n* = 10) obtained using IRTM, showing fitted parameters **(a)** *R*_1_ and **(b)** *k*_*f*_, alongside the derived macroparameters **(c)** *R*_*i*_ and **(d)** *Rk*_3_. Individual subject maps were normalized to the MNI space prior to averaging. The reference efflux rate constant $k_{2}^{{}^{\prime }}$ was fixed to the coupled $k_{2}^{{}^{\prime }}$ value estimated at the VOI level.

Voxel-wise parametric maps (Figure 10) demonstrated high image quality, which was observed even at the individual subject level (see Supplementary Figure 3). The *R_1_* maps exhibited the expected perfusion-weighted contrast, whereas *R_i_* maps additionally reflected information related to [^11^C]CURB trapping. In contrast, *Rk*_3_ maps showed increased susceptibility to noise-related artifacts, particularly in regions of low radiotracer uptake, such as WM.

Linear mixed-effects model [Akaike information criterion (AIC) of − 451, residual standard deviation of 0.02] revealed IRTM-*R_i_* to be a robust predictor of the i2TCM-*R_i_* [β = 0.822, *t*(94) = 17.8, *p* < 0.001]. Importantly, ANOVA analysis resulted in non-significant effects for genotype [*F* (2, 22) = 1.148, *p* = 0.34] and the interaction between IRTM-*R_i_* and genotype [*F* (2, 100) = 0.065, *p* = 0.94], while VOI [*F* (10, 100) = 39.53] was a significant effect (*p* < 0.001). This indicates that the relationship between the IRTM *R_i_* and the respective gold standard estimates remains consistent across AA, AC, and CC cohorts. Additionally, IRTM-derived *Rk*_3_ was also a strong predictor of respective i2TCM measurements [AIC = − 185, residual standard deviation = 0.07, β = 0.862, *t*(94) = 2.9, *p* = 0.005]; however, a significant genotype effect [*F* (2, 14) = 6.42, *p* = 0.011] and IRTM-*Rk*_3_ and genotype interaction [*F* (2, 100) = 5.44, *p* = 0.006] were identified, while VOI effect was non-significant [*F* (10, 100) = 0.87, *p* = 0.564].

## Discussion

Driven by the need to develop a fully non-invasive quantification pipeline for [^11^C]CURB PET, we developed and evaluated IRTM (Figure 1, Equation 10). While FAAH is expressed heterogeneously throughout the brain, complicating the identification of a traditional (ligand-free) reference region, our approach successfully leverages a data-driven WM pseudo-reference region identified via partition-based clustering. Our findings, supported by simulations and validation against gold-standard AIF-based quantification, demonstrate that IRTM provides a robust non-invasive quantification alternative to arterial sampling for studying the endocannabinoid system in humans.

Our simulation analysis revealed systematic biases in IRTM estimates arising from violations of the model assumptions (Salinas et al., 2015), particularly differences in blood volume signal contamination between the target and reference regions. Specifically, errors in *R_i_* were primarily driven by blood volume (Figure 5), whereas *Rk*_3_ was sensitive to both blood volume and λ*k*_3_ levels (Figure 6). Furthermore, the observed increase in *R_1_* bias with its true value suggests a delivery-dependent effect in addition to blood volume effects (Figure 4). Despite these sensitivities, the suitability of using WM as a pseudo-reference region was supported by the increasing concordance between i2TCM and 1TCM fits as cluster location moved progressively toward the center of WM (Figure 7), where the FAAH concentration is lower (Romero et al., 2002; Benito et al., 2007). Our measured WM λ*k*_3_ (0.060 ± 0.022 mL/cm^3^/min) and *K_i_* (0.029 ± 0.007 mL/cm^3^/min) were approximately 2- to 5-fold lower than cortical GM, providing a sufficient signal gradient for the IRTM framework. These findings were corroborated in the validation study, where IRTM-derived *R_i_* values showed robust linear relationship with AIF-based estimates (Figure 8e). Importantly, the model generated high-quality voxel-wise parametric maps, reflecting its parsimonious formulation with only two fitted parameters per voxel (Figure 10).

Although simulations suggested that *R_1_* was less sensitive to blood volume contributions than other simulated parameters, particularly at higher *R_1_* values, its dependency on radiotracer delivery and extraction (i.e., *K_1_*) likely reflects partial mitigation of blood volume effects. In contrast, *Rk*_3_ exhibited a pronounced interaction between the underlying λ*k*_3_ and blood volume, resulting in substantial between-subject variability (Figures 8b,c) and limiting its reliability as a primary quantification metric. The simulated *K_1_* range (0.15 to 0.35 mL/cm^3^/min) represented approximately 91% of values observed in the human datasets, supporting the relevance of the simulation design; however, the bias in *Rk*_3_ is expected to exacerbate for *K_1_* < 0.15 mL/cm^3^/min. Among macroparameters evaluated, *R_i_* emerged as the most robust metric despite the predictable influence of blood volume. Notably, the empirical bias observed in the human data matched the magnitudes predicted by our simulations, with residual variability likely attributable to noise. In fact, simulations incorporating realistic [^11^C]CURB noise characteristics of predicted errors with standard deviation of approximately ± 4% for *R_i_* and ± 9% for *Rk*_3_ (results not shown). It is important to note that a primary objective of this study was to systematically characterize the inherent sources of systematic error in the non-invasive IRTM framework, rather than correcting them at the expense of requiring blood data (i.e., AIF or image-derived input function). An example of this input function dependency can be found in an accompanying analysis using a blood volume-corrected version of IRTM (Supplementary Equation 1), which resulted in a recovery of *R_i_* estimates with respect to AIF-based measurements (see Supplementary material). Thus, our results indicate that IRTM captures the target kinetics while minor systematic bias present in the non-invasive version is a predictable consequence of vascular signal contributions.

To optimize reference region selection, we implemented a partition-based clustering algorithm designed to isolate multiple functional WM subregions. The resulting layered spatial pattern is consistent with varying degrees of GM signal contamination (spill-in). By selecting the centermost cluster (corresponding to the region farthest from cortical and subcortical GM) as reference TAC, we minimize these confounding partial volume effects. The validity of this data-driven selection was further supported by the convergence between i2TCM and 1TCM fits as the clusters approached the WM core. Compared with conventional VOI-based WM TAC extraction, this approach yields a more objective reference TAC. Using this WM cluster as reference within the IRTM framework resulted in excellent predictive association with AIF-based measurements. In addition, the percent standard error of IRTM-derived *R_i_* was comparable to i2TCM-derived λ*k*_3_ and *K_i_* (Table 1), suggesting that our non-invasive method achieves a level of precision similar to the established gold standard.

A key feature of the proposed framework is the coupled estimation of a common $k_{2}^{\prime }$, which reduces the total number of fitted parameters and enhances stability. Analogous to SRTM2, fixing $k_{2}^{\prime }$ based on an initial VOI-level analysis reduced parameter degeneracy in voxel-wise fitting and improved robustness of parametric maps. In addition, IRTM explicitly accommodates irreversible kinetics while retaining a parsimonious parameterization that enables stable estimation at the voxel level. Together, these methodological refinements distinguish IRTM from existing non-invasive approaches and support its use as a practical and robust framework for quantifying FAAH activity with [^11^C]CURB PET. Overall, the present work provides practical guidance for applying the novel IRTM to non-invasive quantification of brain [^11^C]CURB PET data (Figure 3) and highlights the importance of explicitly evaluating model assumptions in each application.

Although results presented here place IRTM as the first fully non-invasive quantification strategy for [^11^C]CURB, some limitations warrant considerations. First, our simulations may not fully capture the range of microparameter combinations encountered in pathology or in pharmacological blocking conditions. For instance, under conditions of high competition with exogenous or endogenous ligands, where the target signal approaches the reference level, *R_i_* may approach unity and lose sensitivity to further binding changes. Additionally, our simulated AIF did not incorporate regional variability in delay and dispersion; however, previous studies suggest that while these effects can impact *K_1_*, their influence on ratio-based parameters is largely mitigated (Mourik et al., 2008; Salinas et al., 2015). Second, the partition-based clustering algorithm implemented here could be refined. For instance, the current range of cluster volumes (9 to 19 mL) could be standardized via a volume-constrained approach to reduce variability in the reference WM VOI volume. In addition, the clustering approach requires careful refining to ensure translation to older or pathological cohorts characterized by cortical atrophy, ventricular enlargement, or WM lesions. Third, and most importantly, the selected WM cluster is a pseudo-reference region containing low but non-zero FAAH expression (Romero et al., 2002; Benito et al., 2007). Indeed, our previously unpublished results from a blocking study using PF-04457845 (Boileau et al., 2015a) demonstrate a 47% reduction in WM standardized uptake value (60 to 90 minutes) following pharmacological blockade, compared to an 88% reduction in GM. While our results show this approach is viable in healthy cohorts, the stability of WM FAAH must be carefully assessed in clinical populations. For instance, neuroinflammatory conditions could lead to an upregulation of FAAH in WM glia (Benito et al., 2007), which would inflate the reference signal and lead to an underestimation of target *R_i_* (Supplementary Figures 4, 5). Future studies involving test-retest reliability and investigating clinical cohorts with altered vascular or glial profiles are essential to characterize disease-specific biases and establish the operational boundary conditions of the IRTM framework. In addition, the implementation of an image-derived input function to correct for blood-borne activity could further refine the accuracy of IRTM.

In conclusion, we have established a robust, fully non-invasive framework for the quantification of FAAH density using [^11^C]CURB PET. For this, we derived IRTM, a novel reference tissue-based model specifically designed for irreversible radiopharmaceuticals. Our validation demonstrates that IRTM maintains strong concordance with gold-standard arterial-based results while its parsimonious parameterization enables stable, high-quality voxel-wise estimation. Combined with an objective, cluster-based approach to select a data-driven WM pseudo-reference region, this framework provides a practical and reliable pipeline for investigating the ECS in the human brain without the need for invasive arterial sampling.

## Data Availability

The datasets presented in this article are not readily available because of ethical and privacy restrictions related to participant confidentiality. Requests to access the datasets should be directed to Isabelle Boileau (Isabelle.Boileau@camh.ca) and Stefan Kloiber (Stefan.Kloiber@camh.ca).

## Footnotes

1
https://www.fil.ion.ucl.ac.uk/spm/
