# Supplementary material for: Quantification of [11C]CURB PET using an irreversible reference tissue model with a cluster-derived pseudo-reference region
Source: Front Neurosci. 2026 Jul 20;20:1882674. doi: 10.3389/fnins.2026.1882674 (PMC13429605; doi:10.3389/fnins.2026.1882674)
Supplement: Supplementary file 1 [file Data_Sheet_1.pdf]

Quantification of [^{11}C]CURB PET using an Irreversible Reference Tissue Model with a Cluster-Derived Pseudo-Reference Region

Lucas Narciso[1–3,*], Shahtaj S. Dheda[1], Raesham Mahmood[1], Rachel F. Tyndale[2,4,5], Tina McCluskey[1], Jerry Warsh[1–3], Bernard Le Foll[1–8], Kimberly L. Desmond[1–3], Stefan Kloiber[2–5], Isabelle Boileau[1–5]

[1] Brain Health Imaging Centre, Centre for Addiction and Mental Health, Toronto, Ontario, Canada
[2] Department of Psychiatry, University of Toronto, Toronto, Ontario, Canada
[3] Institute of Medical Science, Temerty Faculty of Medicine, University of Toronto, Toronto, Ontario, Canada
[4] Department of Pharmacology and Toxicology, University of Toronto, Toronto, Ontario, Canada
[5] Campbell Family Mental Health Research Institute, Centre for Addiction and Mental Health, Toronto, Ontario, Canada
[6] Translational Addiction Research Laboratory, Centre for Addiction and Mental Health, Toronto, Ontario, Canada
[7] Department of Family and Community Medicine, University of Toronto, Toronto, Ontario, Canada
[8] Waypoint Research Institute, Waypoint Centre for Mental Health Care, Penetanguishene, Ontario, Canada

[*] Corresponding Author. lucas.narciso@camh.ca; lucas.narciso@utoronto.ca

SUPPLEMENTARY MATERIAL

SIMULATIONS

Sensitivity to heterogeneity in V_{ND}

Reference tissue models typically rely on the assumption of a uniform non-displaceable volume of distribution ($V_{ND} = K_1/k_2$) across all brain regions. In turn, this assumption justifies the mathematical coupling of the clearance parameter $k_2 = R_1 k_2'$ to maintain parameter identifiability within the IRTM framework. However, if the uniformity assumption is violated, the optimization algorithm may artificially absorb errors associated with V_{ND} heterogeneity into the fitted parameter k_f. For highly lipophilic radiotracer such as [^{11}C]CURB, the WM pseudo-reference region can potentially exhibit higher V_{ND} relative to GM.

To systematically evaluate the impact of regional V_{ND} tissue differences, we simulated target tissue TACs using uniform baseline kinetics, while the reference TAC was generated with varying degrees of reference-to-target V_{ND} mismatch. For this, the simulated target TACs were generated with the microparameter K_1 set to 0.25 mL/cm^3/min (R_1 = 5; K_1' = 0.05 mL/cm^3/min), $k_2 = R_1 k_2'$ ($k_2' \approx 0.0185$ min^{-1}), and k_3 values of 0.038, 0.055, and 0.073 min^{-1}, resulting in theoretical λk_3 values of 0.10, 0.15, and 0.20 mL/cm^3/min. The reference TACs were computed using mismatched k_2' ($k_{2,m}'$) computed as $k_{2,m}' = k_2'/\beta$, where β ranged from 1.00 (uniform V_{ND}) to 1.50 (50% V_{ND} mismatch). Blood volume effects were excluded from this sensitivity analysis.

The simulated impact of reference-to-target V_{ND} heterogeneity on parameter recovery is shown in Supplementary Figure 4. Systematic underestimation in all investigated parameters (R_1, k_f, R_i, and Rk_3), with the magnitude of the bias increasing with the degree of reference-to-target V_{ND} mismatch. Among these parameters, R_1 was the least sensitive to V_{ND} heterogeneity (error < 10% across the simulated range of β). Although the bias in k_f was estimated to be approx. − 20% when reference V_{ND} is 20% higher than target V_{ND}, corresponding R_i and Rk_3 errors were substantially smaller (approx. − 3% for a λk_3 of 0.15 mL/cm^3/min). Interestingly, the bias in both R_i and Rk_3 decreased with increasing k_3, with negligible bias observed for Rk_3 (< 2%) across the range of β when λk_3 was 0.20 mL/cm^3/min.

Effects of violating the 1TCM assumption in the reference region

IRTM assumes that the reference region follows 1TCM kinetics; however, the reference region used in this study (i.e., WM) is known to contain low levels of FAAH. Additionally, disease-related alterations in FAAH expression could violate this assumption and introduce quantification bias. To assess the impact of violating the 1TCM assumption in the reference region, we simulated target tissue TACs using baseline kinetics, while the reference TAC was generated with progressively increasing values of k_3'.

For this, the simulated target TACs were generated with the microparameter K_1 set to 0.25 mL/cm^3/min (R_1 = 5; K_1' = 0.05 mL/cm^3/min), $k_2 = R_1 k_2'$ ($k_2' \approx$ 0.0185 min^{-1}), and k_3 values of 0.038, 0.055, and 0.073 min^{-1}, resulting in theoretical λk_3 values of 0.10, 0.15, and 0.20 mL/cm^3/min. Reference TACs were generated with non-zero k_3' values, yielding target-to-reference k_3 ratios ranging from 1 (i.e., $k_3 = k_3'$) to 10 ($k_3 = 10k_3'$). Blood volume effects were excluded from this analysis. Since the IRTM formulation expects a 1TCM reference region, the optimization routine utilizes an apparent k_2' (k_{2a}'), rather than the true k_2'. In the presence of k_3', k_{2a}' can be approximated by a combination of k_2' and k_3': $k_{2a}' \approx k_2'/(1 + k_3'/k_2')$. Therefore, we obtained k_{2a}' by first fitting each simulated reference TAC to the 1TCM equation and used the resulting k_{2a}' in the IRTM fitting routine.

Based on our simulations, the bias in IRTM parameter increases as the target-to-reference k_3 ratio approaches unity (Supplementary Figure 5). Among them, k_f was the most sensitive to k_3', with errors ranging from – 50% to – 36% for a target-to-reference k_3 ratio of 3 across the simulated range of λk_3. However, corresponding R_i and Rk_3 bias was substantially smaller, ranging from – 11% to 0%. Like our previous simulations, errors in IRTM-derived parameters decreased with increasing λk_3, suggesting lower target λk_3 are more heavily affected by model violations.

IRTM WITH CBV CORRECTION

The PET signal including the CBV (V_b) contribution can be described by $C_{PET}(t) = (1 - V_b)C_T(t) + V_b C_b(t)$, where $C_b(t)$ is the whole-blood AIF. By following the same steps outlined in the main text, the IRTM equation including an explicit CBV correction is given by Eq. (S1).

$$C_{PET}(t) = \left(\frac{1 - V_b}{1 - V_b'}\right) R_1 \left(C_R'(t) + \frac{k_2' k_3}{k_f} \int_0^t C_R'(u)du - k_2 \frac{k_f - k_2'}{k_f} C_R'(t) * e^{-k_f t} \right) + V_b C_b(t) \quad \text{(S1)}$$

where V_b' is the reference CBV, and $C_R'(t) = C_{PET}'(t) - V_b' C_b(t)$, with $C_{PET}'(t)$ as the reference PET TAC.

Supplementary Figures

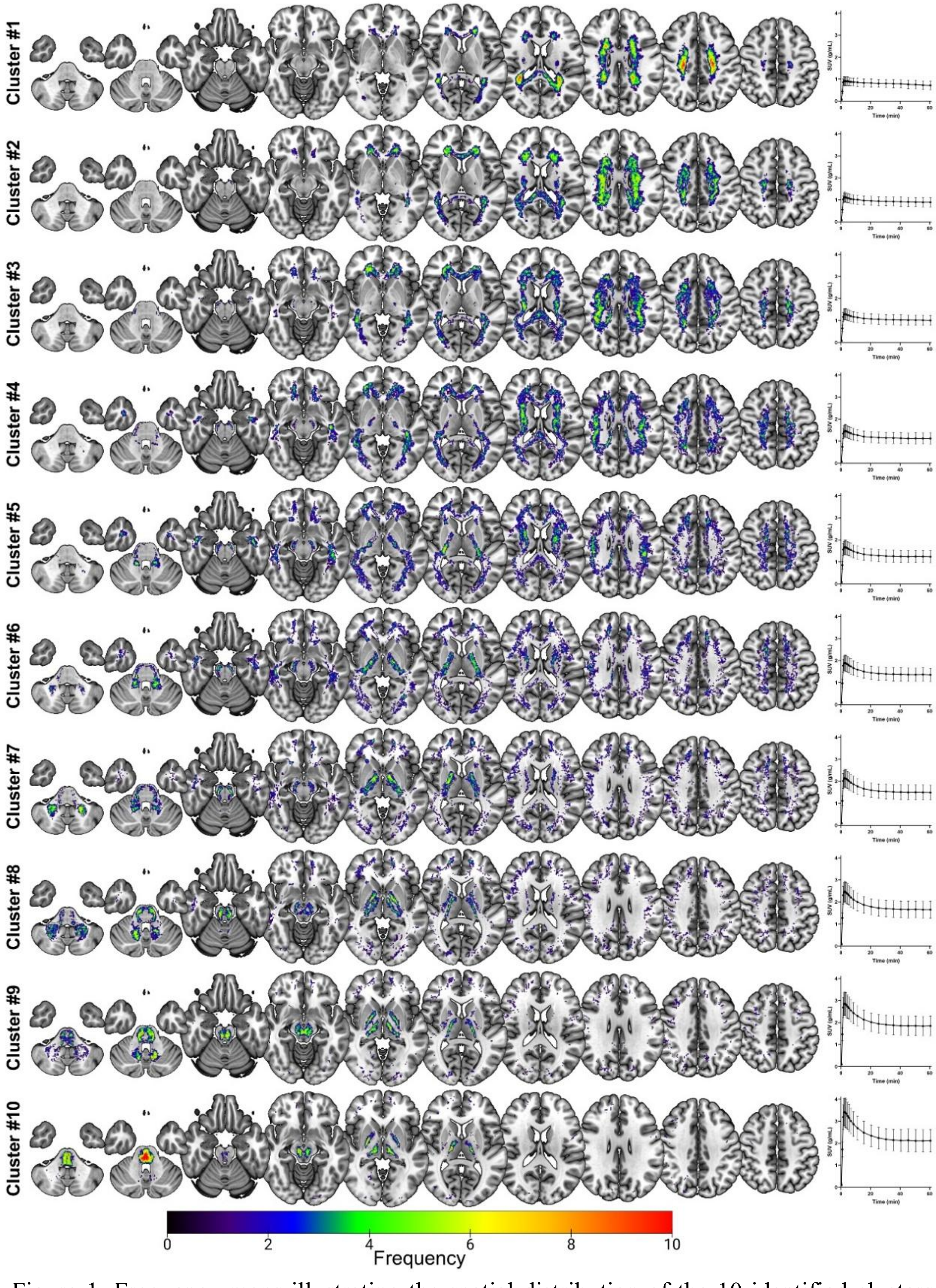

Supplementary Figure 1. Frequency maps illustrating the spatial distribution of the 10 identified clusters. Clusters were ordered spatially for each participant, with Cluster #1 representing the centermost WM region (the reference region). Clusters #2 to #7 are located progressively closer to the GM/WM boundary, while clusters #8 to #10 surround deep GM structures and the brainstem. Notably, the cluster-averaged TACs (right) followed this anatomical gradient, with amplitudes increasing from cluster #1 (lowest) to cluster #10 (highest). Note, the anatomical MRI shown is a template used for visualization purposes only; individual participant anatomy and precise voxel-wise spatial fidelity may differ from this representative image.

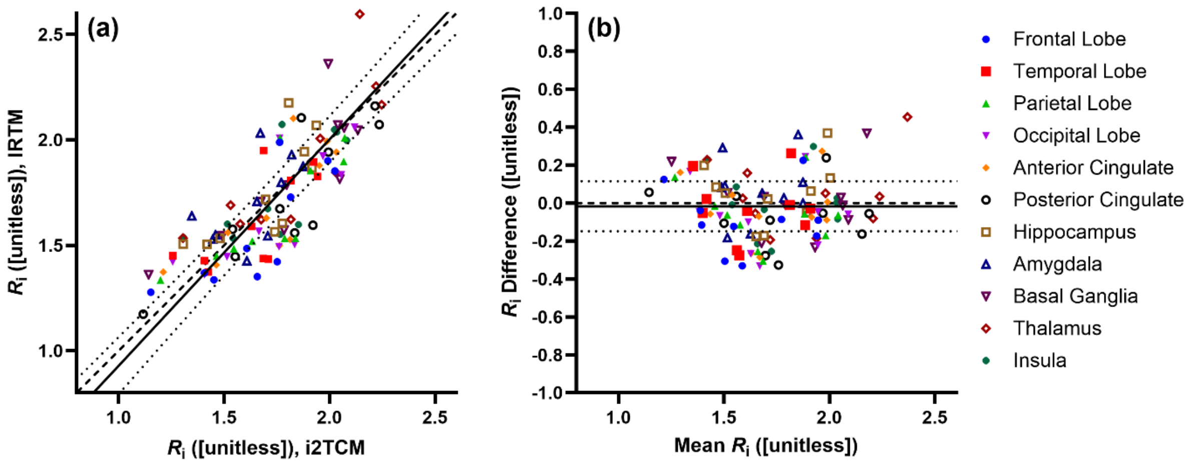

Supplementary Figure 2. Linear regression comparing i2TCM-derived and IRTM-derived R_i estimates (mixed-effects linear regression: $y = 1.07x - 0.15$, $R^2 = 0.95$). Black solid line represents mixed-effects linear regression fit, with dotted lines indicating the ± 95% confidence intervals. Linear regression performed separately for each VOI resulted in mean slope of 0.78 ± 0.07, a mean intercept of 0.36 ± 0.12, and a mean R^2 of 0.61 ± 0.11. Similarly, correlation analysis at the VOI level yielded a mean r of 0.61 ± 0.11, with statistically significant correlations observed for all VOIs except the amygdala. (b) Bland-Altman plots showing the bias in R_i estimated with IRTM relative to AIF-based estimates. Errors in IRTM-derived R_i values ranged from − 5.0% (± 10.4%) for the frontal lobe to 4.5% (± 10.9%) for the amygdala. The dashed black line indicates the mean bias (− 0.017), while the dotted black lines represent the 95% limits of agreement (− 0.148 to 0.115).

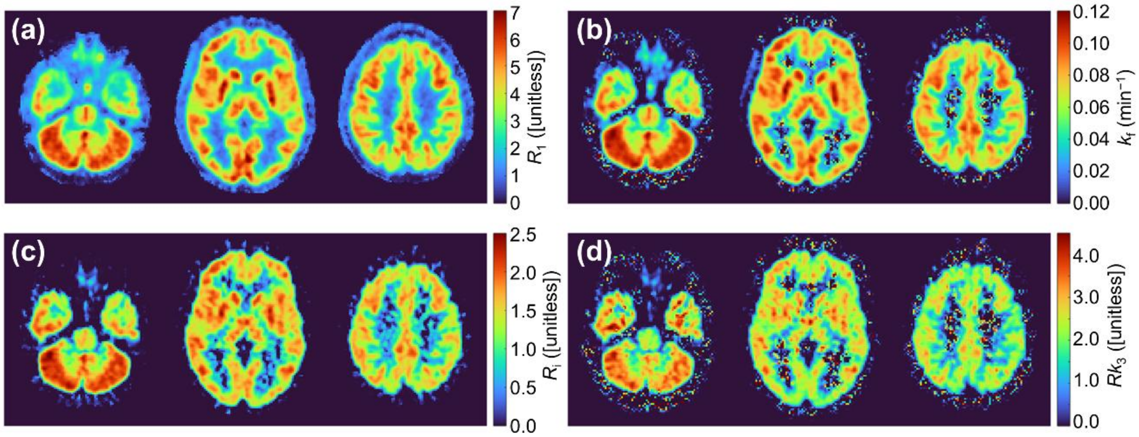

Supplementary Figure 3. Parametric maps from a representative subject obtained using IRTM, showing fitted parameters (a) R_1 and (b) k_f, alongside the derived macroparameters (c) R_i and (d) Rk_3. Maps were normalized to the MNI space. The reference efflux rate constant k_2' was fixed to the coupled k_2' value estimated at the VOI level.

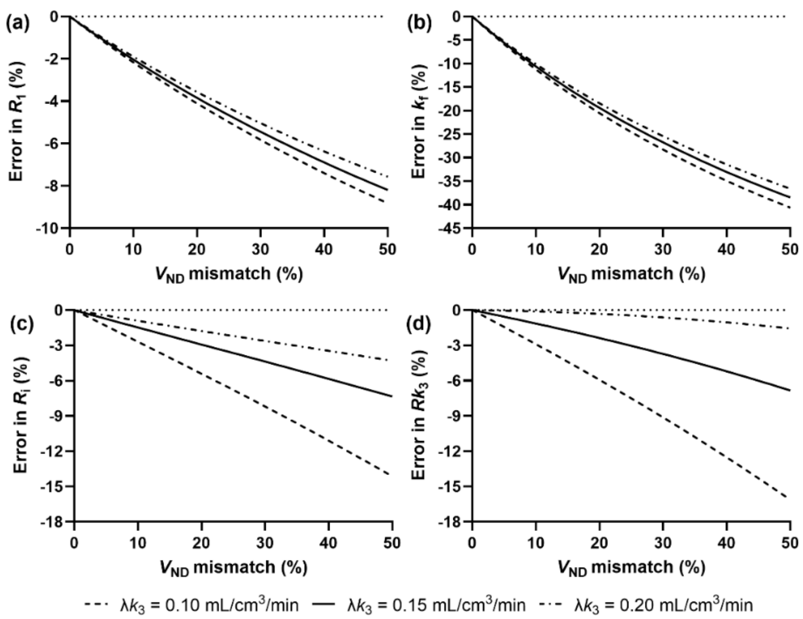

Supplementary Figure 4. Predicted error (%) in (a) R_1, (b) k_f, (c) R_i, and (d) Rk_3 as a function of percent reference-to-target V_{ND} mismatch (β) for theoretical λk_3 values of 0.10 (black dashed line), 0.15 (solid), and 0.20 mL/cm^3/min (dot-dashed). Corresponding K_1 and k_2 values were 0.25 mL/cm^3/min and ~0.0925 min^{-1} ($k_2 = R_1 k_2'$), respectively, with k_3 values of 0.038, 0.055, and 0.073 min^{-1}. The reference TAC was generated with K_1' = 0.05 mL/cm^3/min and using a mismatched k_2' ($k_{2,m}'$) computed as $k_{2,m}' = k_2'/\beta$, where β ranged from 1.00 (uniform V_{ND}) to 1.50 (50% V_{ND} mismatch) and k_2' = 0.0185 min^{-1}. Vascular signal contributions were omitted.

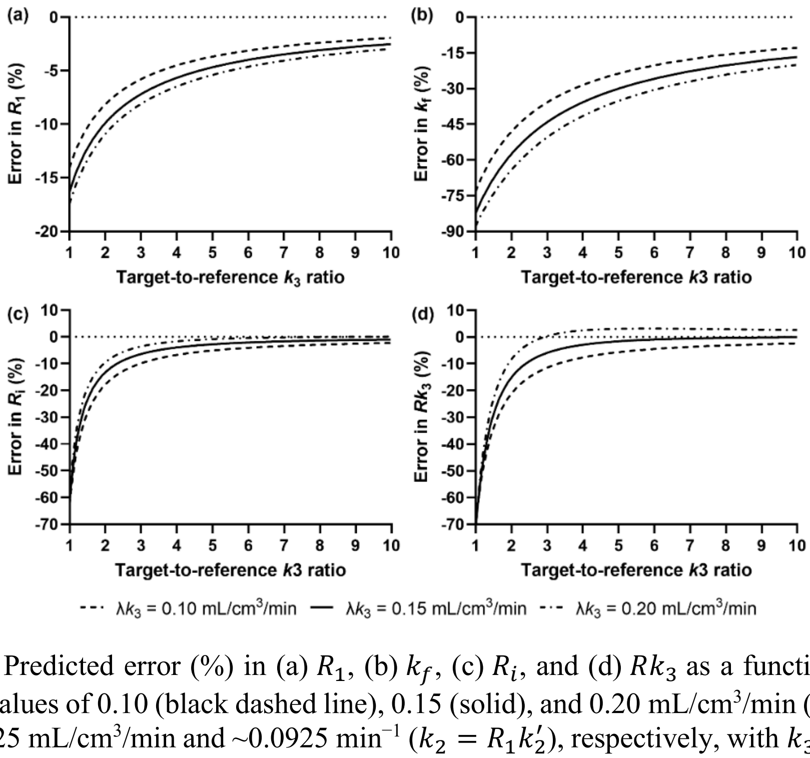

Supplementary Figure 5. Predicted error (%) in (a) R_1, (b) k_f, (c) R_i, and (d) Rk_3 as a function of target-to-reference k_3 ratio for theoretical λk_3 values of 0.10 (black dashed line), 0.15 (solid), and 0.20 mL/cm^3/min (dot-dashed). Corresponding K_1 and k_2 values were 0.25 mL/cm^3/min and ~0.0925 min^{-1} ($k_2 = R_1 k_2'$), respectively, with k_3 values of 0.038, 0.055, and 0.073 min^{-1}. The reference TAC was generated with K_1' = 0.05 mL/cm^3/min, k_2' = 0.0185 min^{-1}, and non-zero k_3' values that yielded target-to-reference k_3 ratios ranging from 1 (i.e., $k_3 = k_3'$) to 10 ($k_3 = 10k_3'$). Vascular signal contributions were omitted.

SUPPLEMENTARY TABLES

Supplementary Table 1. Summary of microparameters obtained with i2TCM and IRTM. Results are expressed in terms of mean ± one standard deviation, alongside the percent standard error (%SE) when applicable.

VOI	i2TCM				IRTM		
	K_1 (mL/cm^3/min) [%SE]	k_2 (min^{-1}) [%SE]	k_3 (min^{-1}) [%SE]	k_f (= $k_2 + k_3$) (min^{-1})	k_f (min^{-1}) [%SE]	k_2 (= $R_1 k_2'$) (min^{-1})	k_3 = $k_f - k_2$ (min^{-1})
**Frontal Lobe**	0.269 ± 0.056 [4.1 ± 1.2]	0.104 ± 0.019 [10.9 ± 4.2]	0.049 ± 0.012 [10.3 ± 4.1]	0.153 ± 0.024	0.066 ± 0.009 [3.4 ± 0.7]	0.041 ± 0.010	0.024 ± 0.004
**Temporal Lobe**	0.232 ± 0.044 [4.4 ± 1.2]	0.089 ± 0.017 [13.2 ± 5.2]	0.051 ± 0.011 [12.1 ± 4.9]	0.141 ± 0.023	0.065 ± 0.009 [3.5 ± 0.7]	0.037 ± 0.009	0.028 ± 0.004
**Parietal Lobe**	0.274 ± 0.051 [4.1 ± 1.2]	0.104 ± 0.018 [11.0 ± 4.2]	0.050 ± 0.011 [10.3 ± 4.1]	0.154 ± 0.023	0.068 ± 0.008 [3.3 ± 0.6]	0.042 ± 0.009	0.026 ± 0.004
**Occipital Lobe**	0.281 ± 0.048 [4.1 ± 1.0]	0.105 ± 0.016 [10.8 ± 3.3]	0.049 ± 0.011 [10.2 ± 3.4]	0.154 ± 0.022	0.069 ± 0.010 [3.3 ± 0.6]	0.043 ± 0.010	0.026 ± 0.004
**Anterior Cingulate**	0.257 ± 0.057 [3.5 ± 1.0]	0.102 ± 0.021 [10.1 ± 4.6]	0.054 ± 0.013 [9.0 ± 4.0]	0.156 ± 0.027	0.068 ± 0.008 [3.3 ± 0.6]	0.040 ± 0.009	0.028 ± 0.004
**Posterior Cingulate**	0.302 ± 0.060 [3.6 ± 1.0]	0.115 ± 0.021 [9.1 ± 3.1]	0.051 ± 0.012 [8.4 ± 3.1]	0.166 ± 0.027	0.072 ± 0.006 [3.2 ± 0.6]	0.045 ± 0.008	0.027 ± 0.004
**Hippocampus**	0.203 ± 0.043 [5.1 ± 1.3]	0.077 ± 0.016 [17.9 ± 6.6]	0.059 ± 0.013 [15.6 ± 5.8]	0.136 ± 0.022	0.069 ± 0.008 [3.8 ± 0.9]	0.033 ± 0.008	0.035 ± 0.006
**Amygdala**	0.186 ± 0.034 [4.7 ± 1.1]	0.072 ± 0.015 [17.6 ± 7.2]	0.060 ± 0.014 [15.3 ± 6.3]	0.132 ± 0.024	0.067 ± 0.009 [4.2 ± 1.1]	0.031 ± 0.008	0.035 ± 0.005
**Basal Ganglia**	0.285 ± 0.063 [4.0 ± 1.0]	0.094 ± 0.017 [11.3 ± 4.5]	0.047 ± 0.012 [11.0 ± 4.3]	0.140 ± 0.022	0.076 ± 0.010 [3.1 ± 0.6]	0.046 ± 0.012	0.030 ± 0.005
**Thalamus**	0.270 ± 0.053 [3.9 ± 1.0]	0.088 ± 0.016 [11.4 ± 4.1]	0.049 ± 0.013 [10.8 ± 4.0]	0.137 ± 0.023	0.076 ± 0.011 [3.1 ± 0.6]	0.044 ± 0.010	0.033 ± 0.008
**Insula**	0.250 ± 0.049 [3.9 ± 0.8]	0.093 ± 0.019 [11.5 ± 4.0]	0.053 ± 0.013 [10.4 ± 3.5]	0.146 ± 0.027	0.070 ± 0.009 [3.2 ± 0.6]	0.040 ± 0.010	0.031 ± 0.004
**WM (Reference Region)**	0.059 ± 0.010 [4.1 ± 1.6]	0.037 ± 0.012 [14.9 ± 7.6]	0.038 ± 0.016 [14.3 ± 9.2]	0.075 ± 0.026	-	-	-

Supplementary Table 2. Summary of results obtained with IRTM including a blood volume correction (Eq. (S1)). Average WM coupled-k_2' was 0.010 ± 0.002 min^{-1} (standard error = 5.5 ± 1.2%). Results are expressed in terms of mean ± one standard deviation, alongside the percent standard error (%SE) when applicable.

VOI	R_1 ([unitless]) [%SE]	Rk_3 ([unitless]) [%SE]	R_i ([unitless]) [%SE]
**Frontal Lobe**	3.95 ± 0.29 [3.0 ± 0.9]	2.69 ± 0.77 [10.8 ± 2.4]	1.57 ± 0.27 [6.0 ± 1.7]
**Temporal Lobe**	3.56 ± 0.27 [3.2 ± 0.9]	3.07 ± 0.80 [12.3 ± 2.6]	1.62 ± 0.23 [6.0 ± 1.5]
**Parietal Lobe**	4.06 ± 0.24 [3.0 ± 0.9]	2.89 ± 0.75 [10.2 ± 2.2]	1.66 ± 0.25 [5.5 ± 1.5]
**Occipital Lobe**	4.16 ± 0.27 [3.0 ± 0.9]	2.84 ± 0.74 [9.9 ± 2.0]	1.66 ± 0.27 [5.4 ± 1.4]
**Anterior Cingulate**	3.79 ± 0.36 [3.1 ± 0.9]	3.16 ± 0.89 [11.3 ± 2.6]	1.69 ± 0.26 [5.5 ± 1.6]
**Posterior Cingulate**	4.35 ± 0.34 [2.9 ± 0.9]	2.96 ± 0.88 [9.4 ± 2.6]	1.73 ± 0.33 [5.2 ± 1.9]
**Hippocampus**	3.24 ± 0.30 [3.3 ± 1.0]	3.98 ± 1.16 [14.2 ± 3.3]	1.75 ± 0.25 [5.5 ± 1.5]
**Amygdala**	3.01 ± 0.30 [3.5 ± 0.9]	4.08 ± 1.05 [15.9 ± 3.8]	1.70 ± 0.20 [5.7 ± 1.4]
**Basal Ganglia**	4.42 ± 0.38 [2.9 ± 0.8]	3.26 ± 0.94 [9.1 ± 1.9]	1.83 ± 0.30 [4.7 ± 1.3]
**Thalamus**	4.24 ± 0.29 [2.9 ± 0.9]	3.57 ± 1.17 [9.6 ± 2.1]	1.89 ± 0.35 [4.6 ± 1.3]
**Insula**	3.82 ± 0.27 [3.1 ± 0.9]	3.40 ± 0.92 [11.0 ± 2.1]	1.77 ± 0.24 [5.1 ± 1.2]
